# A homogeneous immunoassay technology based on liposomes and the complement system enables one-step, no-wash, rapid diagnostics directly in serum

**DOI:** 10.1007/s00216-025-05882-4

**Published:** 2025-05-02

**Authors:** Kilian Hoecherl, Simon Streif, Clemens Spitzenberg, Simone Rink, Arne Behrent, Ferdinand Holzhausen, Christian Griesche, Cornelia Rogoll, Maximilian Foedlmeier, Anna Gebhard, Kacper Kulikowski, Nicole Schaefer, Diana Pauly, Antje J. Baeumner

**Affiliations:** 1 Institute of Analytical Chemistry, Chemo- and Biosensors, University of Regensburg, Universitätsstraße 31, 93053 Regensburg, Germany; 2 Department of Orthopaedic Surgery, Experimental Orthopaedics, Center for Medical Biotechnology (ZMB/Biopark1), University of Regensburg, Universitätsstraße 31, 93053 Regensburg, Germany; 3 Experimental Ophthalmology, University of Marburg, Baldingerstraße, 35043 Marburg, Germany

**Keywords:** Liposomes, Homogeneous immunoassay platform, High-throughput screening, Antibody detection, Complement system

## Abstract

Liposomes are a well-established carrier and controlled release system in medicine and bioanalysis. Their biomimetic capabilities are harnessed for the development of a reliable homogeneous assay platform technology that lends itself to high-throughput screening and point-of-care applications since no wash or separation steps are needed. It was developed for fluorescent, chemiluminescent, and electrochemical detection strategies and applied to antibodies directed against small or polymeric molecules and peptides as model analytes. The simplicity of the approach is achieved as mere binding of analytes or analyte-associated entities to the liposome surface leads to the activation of the complement system, which in turn lyses the liposomes. Released encapsulated marker molecules are quantified and directly correlated to the analytes. Control over the liposome chemistry, including cholesterol content, surface chemistry, and encapsulants, was identified to be key to ensure their general serum and storage stability (more than 40 months at 4 °C and up to 4 weeks at 37 °C) and their efficient and specific response to complement activity. Additional assay conditions of relevance included the concentration of liposomes and their ratio to serum proteins, the amount of complement trigger per liposome, and the activity of complement proteins. Understanding and being able to control the liposomes enable various analysis strategies including the quantification of analytes, determination of complement activity, and evaluation of the therapeutic application potential of antibodies. A time-resolved version of the assay even allows the study of the complex actions of the complement system.

**Graphical Abstract:**

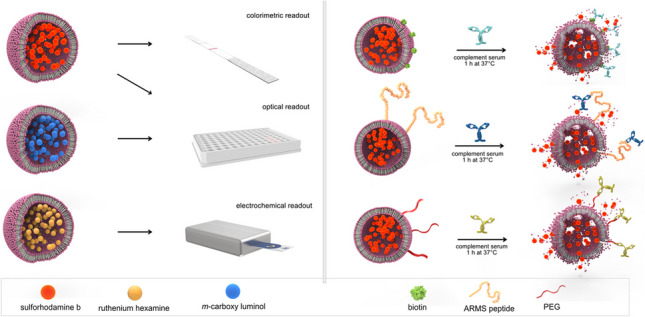

**Supplementary Information:**

The online version contains supplementary material available at 10.1007/s00216-025-05882-4.

## Introduction

Clinical chemistry relies for the most part on the analysis of serum components, as it contains proteins and peptides (globulins, lipoproteins, enzymes, and hormones), carbohydrates, lipids, electrolytes, and other small organic molecules [1–3]. Currently, hundreds of immunoassays are used in clinical laboratories on a daily basis for the diagnosis of various diseases [4] and the development of more sensitive, faster, and simpler methods is of great importance to reduce costs and continue advancements for improved clinical diagnostics. Automation and homogeneous assay formats have already simplified and sped up immunoassay procedures. For example, different detection strategies, such as fluorescence polarization [5], chemiluminescence [6], light scattering methods (turbidity and nephelometry) [7, 8], and EMIT (enzyme multiplied immunoassay technique) or CEDIA (cloned enzyme donor immunoassay) technologies [9, 10], were established. Interestingly, while liposomes are well described in the analytical scientific literature and are broadly applied in drug delivery for medical and cosmetic purposes, they are seldom used in routine settings. Liposomes are lipid bilayer vesicles, which were first described by Bangham et al. [11] in 1965. Their structure enables the encapsulation of hydrophilic or hydrophobic substances in the aqueous cavity or the lipid bilayer, respectively. Through the functionalization of the phospholipids, the liposome surface can be easily modified with any kind of recognition element, such as proteins [12], antibodies [13], DNA [14, 15], peptides [16], and small molecules [17]. Therefore, they have evolved to play an essential role in the pharmaceutical [18], food [19], agricultural [20], and cosmetic industry [21] as carrier systems for the protection and delivery of molecules [22]. Liposomes are particularly important in drug delivery [18] and received a lot of attention through their use as a platform for the COVID- 19 vaccine [23]. In bioanalysis, they are used as signal amplification means by entrapping large quantities of marker molecules, for instance, fluorescent, chemiluminescent, and electrochemically active compounds, or enzymes [12, 14, 15, 24]. Already in the 1970 s and 1980 s, the interaction of the human complement system with liposomes as biomimetics was studied [25–27], whereas today, a commercially available assay uses enzyme-loaded liposomes to investigate the classical pathway of the complement system [28]. The complement system is an important part of our innate immune system and consists of more than 50 plasma and cell surface proteins [29]. Its reaction cascade can be triggered by various means, among them specific surface moieties of foreign cells, ultimately leading to their lysis. Molecules triggering the complement system include carbohydrates, lipopolysaccharides, and antibodies [30]. Taking advantage of liposomes as cell biomimetics, we designed a general assay platform technology to quantify antibodies present, for example, in serum samples. The C1q protein of the complement system binds to antigen–antibody immunocomplexes, in particular the Fc-fragment of antibodies [30]. Studies suggest that at least two antibodies are required on a membrane surface to recruit C1q [31] indicating that surface coverage of antibodies on a liposome surface is a critical factor in assay design. C1q binding facilitates further recruitment and cleavage of several subsequent complement proteins. In the end, the protein C5b allows the association of complement proteins C6–9, enabling the attachment of the complex to the surface and penetration of the lipid bilayer. This leads to the formation of the membrane attack complex (MAC) [30], which forms 10-nm-wide pores in the membrane [32], resulting in the release of encapsulated compounds.

By covalently binding antigens to the liposome surface, the binding of their antibodies can hence be monitored through the formation of the MAC and release of encapsulated marker molecules present within the liposome cavity. Here, we present the proof-of-principle of the platform technology with optical or electrochemical readout. Ruthenium hexamine-loaded liposomes were analyzed with screen-printed carbon electrodes. Chemiluminescent *m*-carboxy luminol and fluorescent sulforhodamine B (SRB) were quantified in microtiter plate (MTP) assays through their chemiluminescent and fluorescent signals, respectively. In the case of electrochemical (EC) and chemiluminescent (CL) detection, the lipid bilayer shields the encapsulants from reaction with the electrode or catalysts, respectively. In the case of fluorescence detection, SRB self-quenches inside the liposomes, resulting in only a minimal fluorescent signal of intact liposomes. This platform therefore offers a novel immunoassay strategy toward the detection of various analytes in serum, demonstrated here using antibodies binding to small and polymeric antigens and peptides as model analytes.

## Materials and methods

### Chemicals and consumables

All chemicals were of analytical reagent grade. The phospholipids 1,2-dipalmitoyl-*sn*-glycero- 3-phosphocholine (DPPC), 1,2-dipalmitoyl-*sn*-glycero- 3-phospho-(1′-rac-glycerol) (sodium salt) (DPPG), 1,2-dimyristoyl-*sn*-glycero- 3-phosphoethanolamine-*N*-[methoxy(polyethylene glycol)− 2000] (ammonium salt) (DMPE-PEG2000), 1,2-dipalmitoyl-*sn*-glycero- 3-phosphoethanolamine-*N*-(biotinyl) (sodium salt) (DPPE-biotin) and the extruder set were purchased from Avanti Polar Lipids (Alabaster, AL, USA); 1,2-dimyristoyl-*sn*-glycero- 3-phosphoethanolamine-*N*-[biotinyl(polyethylene glycol)− 2000] (DMPE-PEG2000-biotin) was purchased from Nanocs Inc. (USA); 1,2-dipalmitoyl-*sn*-glycero- 3-phosphoethanolamine-*N*-(glutaryl) (sodium salt) (*N*-glutaryl-DPPE) from Coatsome; Cholesterol (≥ 99%, C8667), *N*-hydroxysulfosuccinimide sodium salt (sulfo-NHS) (≥ 98%, 56,485), hemin, hydrogen peroxide solution, potassium hexacyanoferrate(II) trihydrate (≥ 98.5%, P9387), hexaamineruthenium(III) chloride (RuHex) (262,005), 2-(2-Methoxyethoxy)ethanamine (901,159; PEG-amine), bovine serum albumin fraction V (BSA), polyclonal goat anti-biotin antibody (B3640), monoclonal rabbit anti-PEG antibody clone RM105 (MABS1214), monoclonal mouse anti-PEG clone 6.3 (MABS1966), Sephadex G- 50, and human complement serum (S1764) were purchased from Sigma-Aldrich/Merck (Darmstadt, Germany); Sulforhodamine B (SRB) (S1307), (1-ethyl- 3-(3-dimethylaminopropyl) carbodiimide-hydrochloride) (EDC) (PG82079), Nunc MaxiSorp high binding microplates (437,111), polyclonal goat anti-rabbit antibody (A16098), polyclonal rabbit anti-mouse antibody (A16162) and polyclonal donkey anti-goat antibody (A16001) were purchased from Thermo Fisher Scientific (Germany); *n*-Octyl-β-d-glucopyranoside (OG) (≥ 98%, CN23), 2-(*N*-morpholino)-ethane sulphonic acid (MES) (≥ 99%, 4259), calcium chloride dihydrate (≥ 99%, 5239) and *N*− 2-Hydroxyethylpiperazine-*N*′− 2-ethane sulphonic acid (HEPES) (≥ 99.5%, HN78) from Carl Roth (Karlsruhe, Germany); DropSens screen-printed carbon electrodes were purchased from Metrohm Germany; *m*-carboxy luminol (purity: 73.6 ± 2.8 wt%) was custom-made by Taros Chemicals GmbH & Co. KG (Germany). Pooled human complement sera (batches 31,758, 33,478, 35,577, 41,174 and 45,270) were purchased from Innovative Research (Novi, MI, USA); ARMS peptide (peptide sequence: IHTELCLPAFFSPAGTQRRFQQPQHHLTLSIIHTAAR) was purchased from ProteoGenix (France). Anti-ARMS antibody A626 was produced using hybridoma technology as described in [33]. Pooled human serum (PHS) was donated by voluntary donors, anonymized, and pooled before being used (1/6 S1, 1/6 S2, 1/3 S3, 1/3 S4). Streptavidin-coated microplates and custom-made lateral flow test strips (streptavidin test line, anti-FITC control line, CN150 membrane) (LFP- 915) were purchased from Microcoat Biotechnologie GmbH (Bernried, Germany). For additional information on common reagents and buffer compositions (Table S1), see SI.

### Liposome synthesis

Liposomes were prepared as previously described using the reverse-phase evaporation method [34]. Briefly, the encapsulant (SRB, *m*-carboxy luminol or RuHex with NaCl to adjust the osmolality) was dissolved in 4.5 mL 0.02 M HEPES buffer, pH 7.5. Lipids were dissolved in 3 mL chloroform and 0.5 mL methanol and sonicated for 1 min at 60 °C. 2 mL encapsulant solution was added to the dissolved lipids and the solution was sonicated for 4 min at 60 °C. Organic solvents were evaporated at a rotary evaporator (LABOROTA 4001, Heidolph, Germany) at 60 °C by stepwise reduction of pressure (900 mbar for 10 min, 850 mbar for 5 min, 800 mbar for 5 min, 780 mbar for 20 min). The solution was vortexed for 1 min, another 2 mL of encapsulant was added, and the solution was vortexed again for 1 min. The residual organic solvents were evaporated at 60 °C (750 mbar for 20 min, 600 mbar for 5 min, 500 mbar for 5 min, 400 mbar for 20 min). The solution was then extruded using polycarbonate membranes with pore sizes of 1 μm, 0.4 μm, and 0.2 μm to obtain unilamellar liposomes. Extrusion was conducted at 60 °C by repeatedly pushing the solution through the syringes (21 repetitions for each pore size). Excess encapsulant was removed by size exclusion chromatography using a Sephadex G- 50 column, followed by dialysis against HSS or CBS buffer in a dialysis membrane Spectra/Por© 4 (MWCO: 12–14 kDa).

### Liposome characterization

The phospholipid concentration of liposomes was determined by inductively coupled plasma optical emission spectroscopy (ICP-OES) measurements (SPECTROBLUE TI/EOP from SPECTRO Analytical Instruments GmbH, Kleve, Germany). Phosphorus was detected at a wavelength of 177.495 nm. Calibration of the device was performed using phosphorus standard solutions between 0 and 100 µM in 0.5 M HNO_3_. The device was re-calibrated before each measurement using 0 and 100 µM standard solutions. Liposome stock solutions were diluted 1:100 or 1:150 in 0.5 M HNO_3_ to determine their total phosphorus content. The total lipid concentration (total lipids) was calculated from the lipid composition used during synthesis.

The hydrodynamic diameter, polydispersity index (PDI) and ζ-potential of liposomes were determined by dynamic and electrophoretic light scattering (DLS, ELS) using a Malvern Zetasizer Nano-ZS (Malvern Panalytical, Germany). Liposome stock solutions were diluted 1:100 in HSS buffer (dispersant refractive index: n_D_^20^ = 1.34; dielectric constant: ε = 78.5; viscosity: η = 1.1185 mPa s). Polymethyl methacrylate (PMMA) semi-micro cuvettes (Brand, Germany) were used for size determination with an angle of 173° and backscattering mode after equilibration for 15 s at 25 °C in three measurement runs with 13 single measurements each. ζ-potential measurements were carried out in folded capillary cells (Malvern Panalytical, Germany) after equilibration at 25 °C for 60 s in four measurement runs with each twenty single measurements.

SRB-encapsulating liposomes were further characterized to determine the maximum fluorescence and liposome stability. Therefore, the fluorescence of lysed (addition of a detergent: 30 mM *n*-Octyl-β-d-glucopyranoside) and intact liposomes (1 µM total lipids in HSS) was measured with a BioTek SYNERGY neo2 fluorescence reader (λ_ex_ = 560 nm, λ_em_ = 585 nm, BW = 10 nm, gain 100). The initial fluorescence was calculated as the ratio of the fluorescence intensities of intact and lysed liposomes.

### Surface modification of liposomes with ARMS peptide

The ARMS-peptide was coupled to carboxyl groups present on the liposome surface using EDC/sulfo-NHS chemistry. The liposome surface was activated using EDC and sulfo-NHS (both 10 mg/mL in 0.1 M MES buffer pH 6) for 1 h at room temperature (RT) and 300 rpm. A 1:100:180 ratio of carboxyl groups:EDC:sulfo-NHS was used. The respective amount of ARMS peptide (0.5 mol%) was added, and the solution was further incubated for 3 h at RT and 300 rpm. The reaction was quenched through the addition of PEG-amine (161 eq. per carboxyl group) and the solution was incubated for another 30 min at RT and 300 rpm. The peptide-modified liposomes were dialyzed overnight against HSS buffer in a Spectra-Por® Float-A-Lyzer® G2 (1 mL, MWCO: 1000 kDa) to remove excess coupling reagents. The total lipid concentration was again determined by ICP-OES measurement.

### Homogeneous SRB-liposome complement assay

The homogeneous complement-dependent assay contains four conditions, each in triplicates: liposomes in liposome complement buffer (LCB; negative control), liposomes in active complement serum (aS) or in inactive complement serum (iaS; negative control) and lysed liposomes as positive control using a detergent (30 mM OG + aS). The complement serum was inactivated by the addition of an inactivation complement buffer (iaCB, containing 0.2 M EDTA and 0.5 µM EGTA). Active complement serum is also added to the positive control to account for the serum-enhanced fluorescence. LCB, sucrose in LCB (0.2 M per well), iaCB, and 300 mM OG were added to a black, flat-bottom MTP on ice to prevent complement activation before the start of the measurement. Liposomes were diluted, if required, incubated with antibodies for 1 h at RT and 300 rpm before being added to the MTP. Finally, complement serum was added to the aS, iaS, and OG samples. The fluorescence was measured in 1.5 min intervals for the first 15 min, followed by 5 min intervals for another 45 min. Fluorescence measurements were performed three consecutive times with a BioTek SYNERGY neo2 fluorescence reader (λ_ex_ = 565 nm, λ_em_ = 585 nm, BW = 5 or 8 nm, gain 150).

### LFA complement assay

Similar to the MTP-based complement assay, the following samples were prepared: LCB, iaS, aS, and 30 mM OG + aS in triplicates. Serum was inactivated by mixing 2.5:1 with inactivation complement buffer. Liposome samples were incubated for 1 h at 37 °C. After incubation, aS was also added to the LCB sample, as serum proteins influence the migration behavior on the LFA test strips. SRB-liposome solutions were diluted 1:1 in the respective running buffers and added to a clear 96-well MTP (50 µL and 50 µM total lipids). Test strips were placed into the wells, and the respective washing buffer (100 µL) was added after 5 min. Pictures were taken after 20 min using a Canon EOS 550D camera with a Canon EFS 18–55 mm lens from a distance of 15 cm, with consistent lighting and the following settings: ISO 100, aperture 3.5, exposure time 1/30 s, focal length 18 mm, and white balance daylight (5200 K). The raw files were analyzed using ImageJ [35]. The color channels were split, and the background of the green channel was subtracted for background smoothing (50 pixels) before black/white inversion. The brightness was adjusted to make the lines visible for the naked eye. The intensities of the background, test, and control line were measured threefold using a rectangle of 80 × 45 pixels. The average intensities were calculated, and the background signal was subtracted.

### Heterogeneous binding assay

Antibodies (2 µg/mL in PBS, 100 µL) were immobilized in a high binding MTP overnight at 4 °C. The solution was removed, and the plate was blocked with BSA (1 w/v% in PBS-T, 150 µL) for 1 h at RT and 300 rpm. When using secondary antibodies, the MTP was washed three times with PBS (150 µL each) before the addition of the primary antibody (2 µg/mL in PBS, 100 µL) and incubation for 1 h at RT and 300 rpm. The MTP was washed twice with PBS-T or PBS and three times with HSS (each 150 µL) before the addition of SRB-liposomes (10 µM total lipids in HSS, 100 µL) and incubation for 3 h at RT and 300 rpm. The plate was washed three times with HSS (150 µL) and bound liposomes were lysed by the addition of 30 mM OG in double-distilled water (100 µL; 15 min incubation at RT and 300 rpm). The fluorescence was measured with a BioTek SYNERGY neo2 fluorescence reader (λ_ex_ = 560 nm and λ_em_ = 585 nm, BW = 10 nm, gain 100).

### Heterogeneous *m*-carboxy luminol-liposome complement assay

Heterogeneous complement assays with *m*-carboxy luminol-encapsulating liposomes were performed in streptavidin-coated MTPs. The streptavidin-coated plate was washed with 200 μL Glycine–NaOH buffer (pH 8.6) for 5 min while shaking before incubating 100 μL of the respective biotin-modified liposomes in Glycine–NaOH buffer (50 µM total lipids) overnight. The unbound liposomes were removed, and the MTP was washed three times with 200 μL CBS buffer (pH 10.5) for 5 min. When a goat anti-biotin antibody was tested as a complement trigger, the liposomes were incubated with 100 μL antibody (0.2 mol% in PBS) for 1 h at RT after the liposomes were bound to the MTP and washed twice with 200 μL Glycine–NaOH buffer and once with 200 μL PBS. The bound liposomes were incubated with active or inactive serum in LCB and incubated for 1 h at 37 °C with gentle shaking. After incubation, the MTP was washed three times with 200 μL CBS for 5 min, followed by lysis of liposomes for 5 min while shaking with 100 μL 30 mM OG in CBS. The lysed liposomes were transferred into a white, flat-bottom MTP. The chemiluminescence intensity was measured with a microplate reader after adding 50 μL 40 mM H_2_O_2_ and 50 μL 4 μM hemin in CBS. A blank reading was performed prior to the H_2_O_2_ addition. After the addition of H_2_O_2_, 5 s shaking (425 cpm) was started, and the CL intensity was measured with gain 80, read height (RH) 1 mm, and integration time 2 s.

### Electrochemical RuHex-liposome complement assay

Electrochemical measurements were performed on a DropSens screen-printed carbon electrode (DRP- 110). RuHex-encapsulating liposomes were incubated for 1 h at 37 °C and 300 rpm in the respective samples (LCB, aS, iaS, and 30 mM OG + aS). After incubation, a 2.5 M CaCl_2_ solution was added to adjust the Ca^2+^ content to 100 mM for improved electrochemical detection. 50 µL of each sample was pipetted onto the electrode, and square wave voltammograms were recorded from − 0.5 V to 0.1 V vs Ag, with E_step_ = 4 mV, E_amp_ = 40 mV, and f = 2 Hz. A new electrode was used for each single measurement. The current peaks resulting from the electron transfer of the [Ru(NH_3_)_6_]^3+^/[Ru(NH_3_)_6_]^2+^ redox couple were identified near − 0.21 V.

### Data evaluation and statistical analysis

Raw data from homogeneous SRB-liposome complement assays were processed as follows: The mean fluorescence intensities were normalized to the endpoint fluorescence intensity of the positive control (‘normalized fluorescence intensities’). To obtain the ‘corrected lysis’, which refers to the actual complement lysis, the background signal of the negative control (iaS) was subtracted from both the aS condition and the positive control before normalization.

Data were analyzed statistically using OriginPro 2024 Software. A two-sample independent t-test was performed to compare two groups: the active serum sample and the inactive serum sample (negative control). To compare three or more different samples, a one-way analysis of variance (ANOVA) with a post hoc Tukey test was performed. p-values ≤ 0.05 were considered statistically significant. * *p* ≤ 0.05, ** *p* ≤ 0.01, *** *p* ≤ 0.001, and ns = not significant.

## Results and discussion

A novel liposome-based immunoassay platform utilizing the complement system was developed to enable a simple and rapid detection of antibodies in serum. While the concept of complement-induced lysis and serum stability (stealthiness) of liposomes was studied with fluorescent, chemiluminescent, and electrochemically active encapsulants, the majority of subsequent experiments were performed with fluorescent liposomes only, as they allowed an easier assay procedure and a more sensitive readout. Moreover, it was demonstrated that this platform can also be applied in a simple POC lateral flow assay (LFA) format beyond the intended microplate-based high-throughput screening (HTS) approach. As proof of principle, antibody-triggered complement lysis of liposomes was specifically induced through selected surface functionalizations (biotin, PEG, peptide). In addition, naturally occurring anti-PEG antibodies were detected. Storage stability of selected liposomes was monitored at 4 °C, RT, and 37 °C for up to 40 months to assess colloidal stability, liposome integrity, and serum stability.

### Tuning of liposome serum stability by cholesterol and encapsulant content

**Fig. 1:**
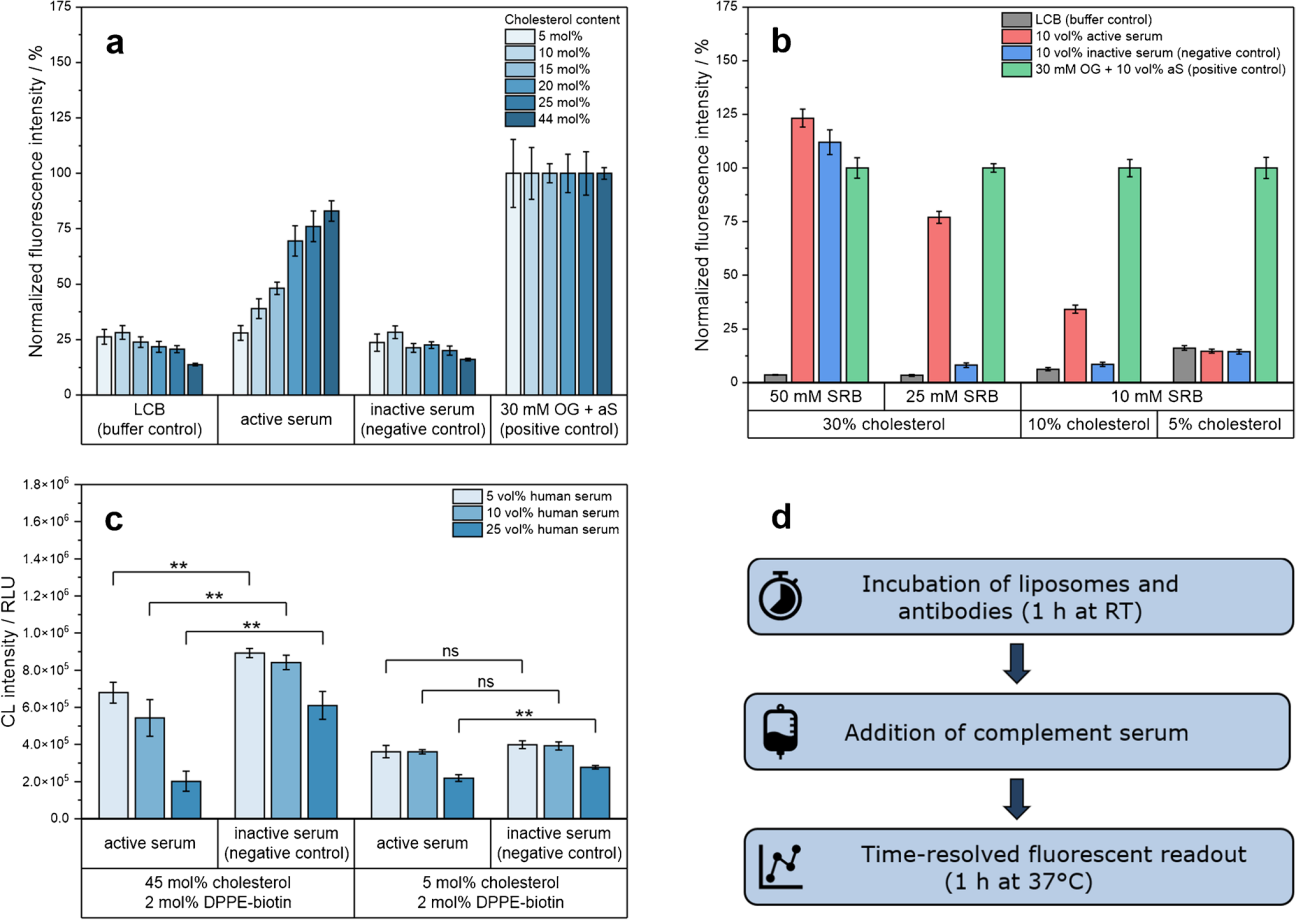
Optimization of the liposome composition to generate stealth liposomes. **a** Homogeneous complement assay of 10 mM SRB-encapsulating liposomes (10 µM total lipids; batches S1–6) showing the cholesterol dependency of liposome stealthiness in active serum (10 vol% PHS). Statistical analysis (two-sample independent t-test) revealed a significant difference between active and inactive serum samples at a cholesterol content of 10 mol% (p = 0.026) and above (p < 0.001). In contrast, no significant difference was observed at a cholesterol content of 5 mol% (p = 0.881). **b** Liposome stealthiness in active serum depending on SRB and cholesterol content (batches S7–10). 10 vol% human serum was used as a complement source (IRS41174). Statistical analysis (two-sample independent t-test) showed no significant difference between active and inactive serum samples of liposomes containing 10 mM SRB and 5 mol% cholesterol (p = 0.714), while in the other cases either a statistically significant amount of complement lysis (p < 0.001) or general serum stability was observed. Fluorescence measurements were carried out for 1 h at 37 °C in LCB, iaS, aS, and 30 mM OG + aS. Fluorescence intensities were normalized to the endpoint fluorescence of the positive control. λ_Ex_ = 565(5 or 8) nm and λ_Em_ = 585(5 or 8) nm; gain 150. T = 37 °C. n = 3. **c** Heterogeneous complement assay of biotin-modified chemiluminescence liposomes (30 mM *m*-carboxy luminol; batches CL1–2). Statistical analysis (two-sample independent t-test) showed a significant difference between active and inactive serum samples of high-cholesterol liposomes. Low-cholesterol liposomes remained stealth and showed no statistical difference in 5 and 10 vol% serum, whereas 25 vol% led to minor lysis in active serum. Liposomes (50 μM total lipids) were immobilized overnight on a streptavidin-coated MTP and incubated for 1 h at 37 °C in either inactive serum or active serum (5, 10, or 25 vol% PHS in LCB). Chemiluminescence measurements were performed by adding 50 μL of 4 μM hemin and 50 μL 40 mM H_2_O_2_ in 0.01 M CBS, pH 10.5, 2 s integration time, gain 80, RH 1 mm, T = 25 °C, n = 3. **d** Flow chart of the homogeneous complement assay procedure for SRB-encapsulating liposomes

Cholesterol is a key regulator of membrane fluidity; it influences the phase transition and rigidity of the lipid bilayer, thereby supporting liposome stability [36]. In previous works, liposomes containing 44 mol% cholesterol have been established as a versatile platform with different marker molecules for various applications, such as detection of SARS-CoV- 2 neutralizing antibodies [12], DNA from *C. parvum* [15] and nucleic acids from Influenza A, Influenza B, and SARS-CoV- 2 [14] employing fluorescent, electrochemiluminescent, or electrochemical readout strategies. However, the use of high-cholesterol liposomes in active complement serum led to complement-induced lysis of liposomes. Cholesterol is a known complement trigger when used in high concentrations in vesicles or as cholesterol crystals [37–40]. In the intended format, non-specific liposome lysis allows false-positive signals, which is why stealth (serum stable) liposomes are required. Therefore, a stepwise reduction of the cholesterol content (5, 10, 15, 20, 25, and 44 mol%) was thoroughly studied using 10 mM SRB-encapsulating liposomes (Fig. 1a). As expected, it was found that the less cholesterol that was used, the less lysis was obtained, and it can be concluded that the cholesterol content can be used to tune the liposome stealthiness in serum. Another crucial parameter for the serum stability of the liposomes was found to be the SRB content. While higher concentrations are desirable to provide a more sensitive readout, it was found that its encapsulation was affected by the cholesterol content (Fig. 1b). While 10 mM SRB-liposomes can easily be formed regardless of the cholesterol content, low-cholesterol concentrations do not support higher SRB concentrations. Specifically, at 5 mol% cholesterol and > 10 mM SRB, encapsulation efficiency goes toward zero (data not shown). Therefore, more than 5 mol% cholesterol is required to entrap 25 mM SRB or higher. Furthermore, SRB, albeit highly water soluble, probably associates with the lipid bilayer [41], which led to undesired interactions with serum, as stably formed liposomes with 50 mM SRB and 30 mol% cholesterol lysed even in inactive serum. Thus, the optimal composition for stealth liposomes, where the interaction with serum components is solely determined by the liposome surface chemistry, was found to be 5 mol% cholesterol and 10 mM SRB. This composition was used for all subsequent studies utilizing biotin-, PEG-, and carboxyl-functionalized lipids to facilitate liposome surface modification. Stealth 5 mol% cholesterol liposomes were also successfully synthesized using chemiluminescent (*m*-carboxy luminol) and electrochemical (ruthenium hexamine (RuHex)) encapsulants (Fig. 1c and Fig. S1a). As expected, 44 mol% cholesterol RuHex and *m*-carboxy luminol-liposomes also triggered complement-induced lysis (Fig. 1c and Fig. S1b) due to the high-cholesterol content. It should be noted that the ratio of liposomes to serum chosen for the RuHex-liposomes led to only 9% lysis (100 µM total lipids to 10 vol% complement serum) and will be discussed further below. In the case of the electrochemical readout, interference from serum components had to be considered when selecting the encapsulant. Among the electroactive components studied, only RuHex allowed selective and sensitive detection in serum (Figs. S2 and S3).

### Proof-of-principle antibodies as external complement triggers and assay design setup

Antibodies are well-known complement triggers of the classical pathway and activate the complement system by binding the complement protein C1q to their Fc fragments [30]. Monoclonal and polyclonal antibodies directed against biotin, PEG, and a peptide were studied as model analytes. Biotinylation and PEGylation of liposomes were accomplished through the addition of functionalized phospholipids during liposome synthesis. The ARMS peptide originates from the age-related maculopathy susceptibility 2 (ARMS2) protein and is highly associated with age-related macular degeneration (AMD) [42]. It was conjugated to carboxyl-bearing liposomes using EDC/sulfo-NHS chemistry. Liposomes were characterized with respect to size, size distribution, ζ potential, and initial fluorescence signal as an indication for agglomeration and colloidal stability (Tables S2–4). Biotin- and PEG-liposomes revealed hydrodynamic diameters ranging from 104 to 135 nm and PDIs between 0.13 and 0.14, indicating colloidal stability. Furthermore, the successful surface modification with biotin and PEG was demonstrated using simple binding assays (Fig. S4), and in the case of ARMS by DLS. It was found that ARMS-modified liposomes tend to agglomerate, which is probably a result of the electrostatic interactions of the negatively charged liposome surface and the positively charged peptide under physiological conditions (pH 7.4).

**Fig. 2:**
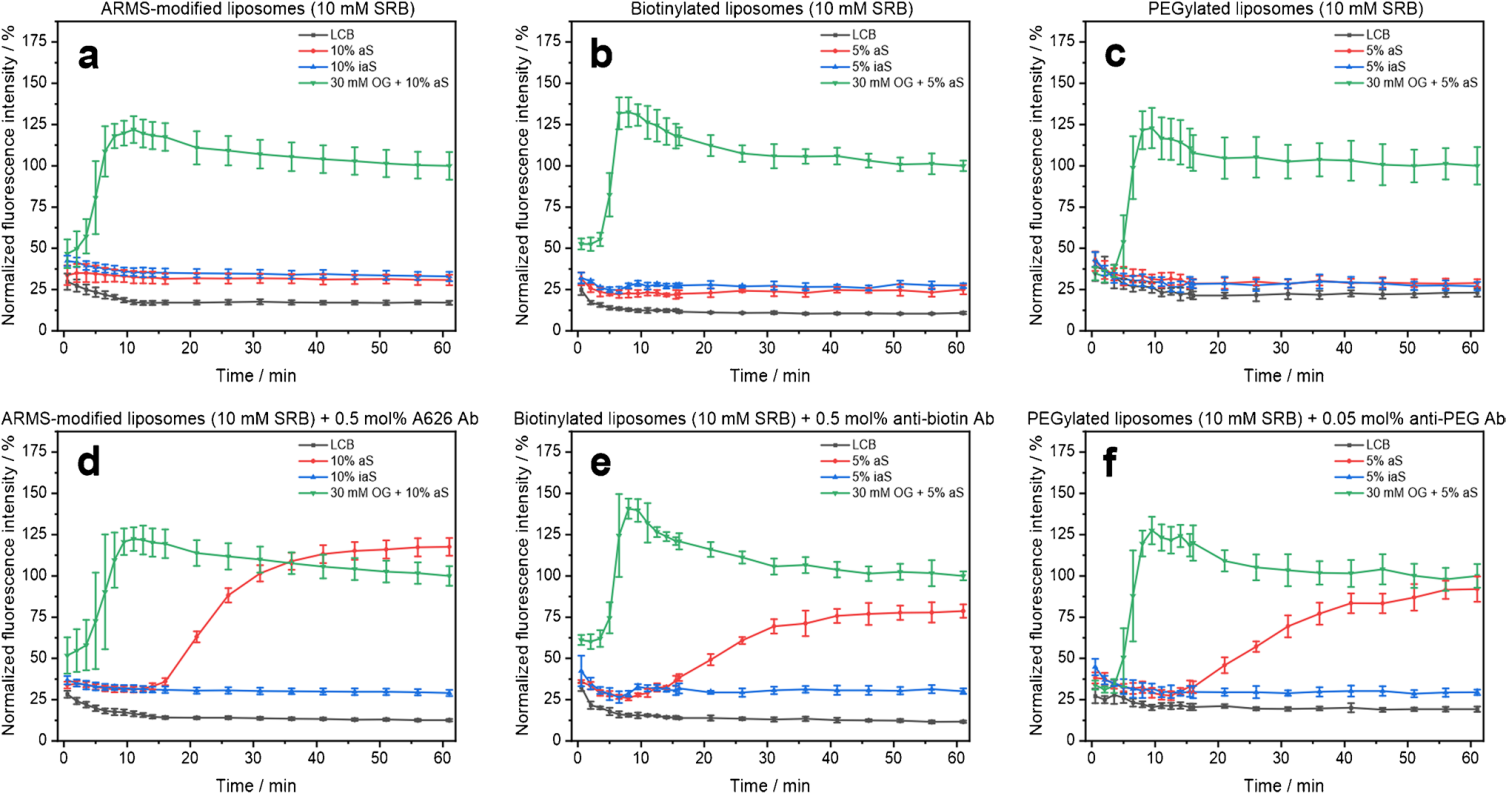
Homogeneous complement assay of 10 mM SRB-liposomes using antibodies as complement trigger. Time-resolved normalized fluorescence intensities of 0.5 mol% ARMS-modified liposomes (10 µM total lipids; batch S11) **a** without or **d** with 0.5 mol% complement-triggering anti-ARMS antibody (A626). ARMS-liposomes were incubated with the A626 antibody for 1 h at 300 rpm. 10 vol% human serum was used as complement source (IRS35577). Time-resolved fluorescence intensities of biotinylated liposomes (1 µM total lipids; batch S12) **b** without or **e** with 0.5 mol% complement-triggering anti-biotin antibody. Biotin-liposomes were incubated with the anti-biotin antibody for 1 h at 300 rpm. 5 vol% human serum was used as complement source (IRS45270). Time-resolved fluorescence intensities of PEGylated liposomes (1 µM total lipids; batch S1) **c** without or **f** with 0.05 mol% complement-triggering anti-PEG antibody (clone RM105). PEG-liposomes were incubated with the anti-PEG antibody for 1 h at 300 rpm in 5 µL HSS. 5 vol% human serum was used as complement source (IRS45270). Fluorescence measurements were carried out for 1 h at 37 °C in LCB, iaS, aS and 30 mM OG + aS and normalized to the endpoint fluorescence of the positive control. λ_Ex_ = 565(8) nm and λ_Em_ = 585(8) nm; gain 150. T = 37 °C. n = 3

**Fig. 3:**
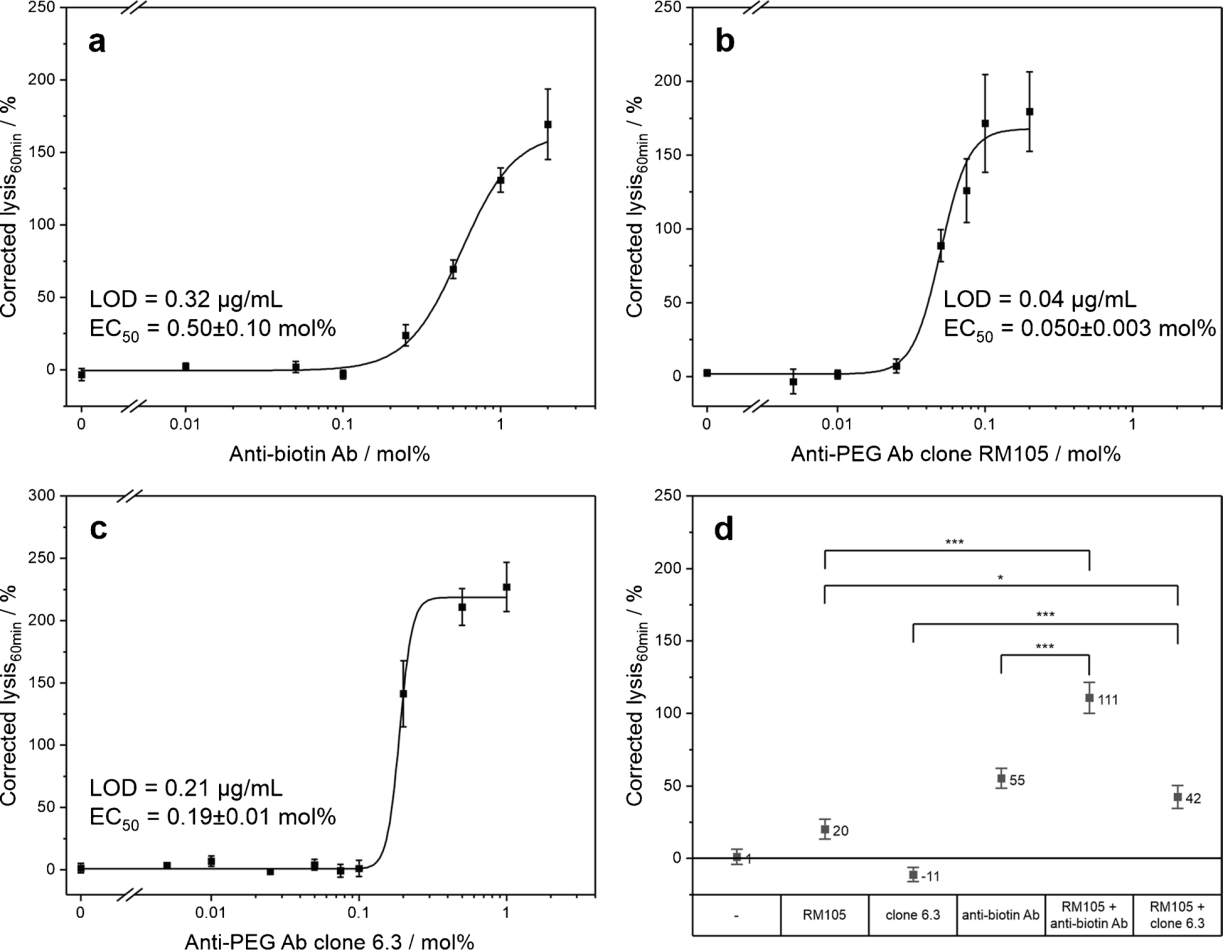
Dose–response curves of liposome lysis dependent on the antibody concentration. Corrected lysis values of **a** Biotin- (batch S12) or **b**,** c** PEG-biotin-liposomes (batch S1) (10 mM SRB, 1 µM total lipids) in a homogeneous complement assay performing an anti-biotin or anti-PEG (clone RM105 or clone 6.3) antibody titration. **d** Different combinations of these antibodies (0.03 mol% clone RM105, 0.1 mol% clone 6.3, 0.3 mol% anti-biotin antibody) with PEG biotin-liposomes (batch S1). A one-way ANOVA, including a post hoc Tukey test, was performed for statistical analysis (p < 0.05) of the antibody combinations. Liposomes were incubated with the respective amount of antibodies for 1 h at 300 rpm. 5 vol% human serum was used as a complement source (IRS45270). Fluorescence intensities were adjusted by the iaS signal and normalized to the endpoint fluorescence of the positive control. λ_Ex_ = 565(8) nm and λ_Em_ = 585(8) nm; gain 150. T = 37 °C. n = 3

The ability of the liposomes to bind to their respective antibodies and the subsequent triggering of the complement system was studied using a time-resolved fluorescence readout. To separate binding and complement activation steps in these preliminary studies, liposomes and antibodies were initially incubated for 1 h prior to serum addition (Fig. 1d). For all modifications, namely ARMS modification (Fig. 2d), biotinylation (Fig. 2e), and PEGylation (Fig. 2f), antibody-triggered complement activation was successfully demonstrated. Liposome lysis started after 15–30 min in the presence of a complement trigger before reaching saturation after 50–60 min, while liposomes remained stealth in the absence of triggering antibodies (Fig. 2a–c). As a positive control, providing 100% liposome lysis, a detergent was added to the liposome, analyte, and serum mixture. As negative controls, either liposomes in complement assay buffer (LCB) or liposomes in inactivated serum were used. Both negative controls serve as indicators of possible liposome instability per se or that caused by serum components. An observed offset of the inactive serum signal to the buffer control is likely due to agglomeration of liposomes with serum proteins and thus enhanced scattering. A similar effect was observed in active serum samples of stealth liposomes (Fig. 2a and b). Most nanoparticles and nanovesicles form a protein corona when in contact with biological fluids. This interaction is influenced by surface charges and overall chemical composition and leads to some minor degree of agglomeration [43, 44]. Not surprisingly, PEGylated liposomes are less likely to agglomerate due to PEG’s shielding effect, which reduces non-specific interactions and thus serum protein adsorption (Fig. 2c) [45]. To minimize agglomeration and avoid further scattering effects, several additives (trehalose, sucrose, D-galactose, lactose, NaCl, and BSA) were tested for their effect on the background signal. Sucrose (200 mM) was the most promising candidate, reducing the background signal by 77% (Fig. S5). In the case of the positive control, general effects on the fluorophores can be observed over the incubation period as fluorescence is highly dependent on the environment, such as the solvent, pH, and temperature. The initial dramatic rise of the signal is attributed to liposome lysis as temperature rises from 4 to 37 °C (Fig. 2). The subsequent minor decrease is likely due to quenching effects of the detergent, which may cause the active serum sample to exceed the positive control. In addition, serum proteins create a more hydrophobic environment, leading to increased fluorescence signals [46]. Consequently, free SRB or lysed SRB-liposomes exhibited 65–141% higher fluorescence intensities in serum than in buffer solutions (Fig. S6). However, the fluorescence-enhancing effect of serum proteins appears to be less pronounced in OG-containing samples, which leads to an even greater discrepancy between the endpoint signal of the positive control and the active serum sample. For the simplified endpoint evaluation, fluorescence intensities were normalized to the endpoint of the positive control, allowing for lysis values exceeding 100% (Figs. 2d and 3). While the assay platform is envisioned to be an endpoint assay, the time-resolved measurement enables precise monitoring and thus a better opportunity for data interpretation throughout the development of the technology.

Antibody-triggered complement lysis was also investigated with *m*-carboxy luminol-liposomes; however, in the case of CL quantification, only endpoint detection is possible. Initial experiments for CL quantification of the liposomes indicated strong interference of the radical-dependent CL reaction due to the radical scavenging nature of serum, leading to an about 64-fold higher limit of detection (data not shown). Moreover, *m*-carboxy luminol undergoes continuous degradation through reactions with naturally occurring H₂O₂ and hemin in serum, depending on the release rate of the encapsulant, which complicates reproducible detection in a homogeneous assay format. Therefore, a heterogeneous format was developed (Fig. S7). Here, biotinylated *m*-carboxy luminol-liposomes were immobilized in an MTP via the biotin-streptavidin interaction. After incubation with anti-biotin antibodies and serum or serum solely, the wells were washed, and the remaining intact liposomes were lysed and quantified. In the presence of complement-triggering anti-biotin antibodies, a lower CL signal was observed in 10 and 25 vol% active serum due to complement-induced liposome lysis (Fig. S8). However, the higher concentration of liposomes or greater steric hindrance of a heterogeneous approach rendered the assay less sensitive than the fluorescence approach.

In the case of the EC liposomes (assay procedure Fig. S9), no antibody-triggered liposome lysis was detected (data not shown). Since complement-induced lysis depends on the liposome to serum ratio, the absence of lysis in the case of EC liposomes is likely due to the higher liposome concentration (100 µM total lipids) required for EC detection. This finding was later confirmed using SRB-liposomes by investigating the ratio between total lipids and serum concentration needed to avoid oversaturation of the complement system with liposome surfaces. This high liposome concentration was required because the EC approach proved to be three orders of magnitude less sensitive than fluorescent detection, with an LOD of 13 µM total lipids for RuHex-liposomes compared to 49 nM for SRB-liposomes. In the future, the EC liposome approach will only be used to study complement activity via high-cholesterol liposomes, rather than antibody quantification via ligand binding.

### Quantification of antibodies and analyzing complement activation potential for therapeutic applications

For the quantification of antibodies, a typical dose–response curve with respect to antibody concentration was recorded to determine the limit of detection and the corresponding EC_50_ values. Two monoclonal antibodies against PEG (clone RM105 and clone 6.3) and a polyclonal antibody against biotin were investigated (Fig. 3a–c). Typical binding curves were recorded to provide reliable and quantifiable data for comparative studies. As mentioned above, specific binding of the antibodies to the fluorescent liposomes was confirmed in a normal heterogeneous binding assay where the antibodies were immobilized via adsorption to the MTP surface and liposomes bound to them via their surface modifications (PEGylation or biotinylation) (Fig. S4). In the case of clone RM105, adsorption worked poorly, and immobilization via a secondary anti-rabbit antibody was chosen instead. Interestingly, the clone RM105 antibody, which is directed against the terminal methoxy group of the PEG chain, also revealed binding to PEGylated liposomes with a terminal biotin moiety, suggesting binding through cross-reactivity toward other PEG structures, in this case, the PEG backbone [47]. As expected, the clone 6.3 antibody, which is directed against the PEG backbone, showed binding to both PEGylated liposomes, and the anti-biotin antibody bound strongly to the biotinylated liposomes. It should be noted that differences in the overall fluorescence signals (Fig. S4) were due to the use of differently sized liposomes with different marker encapsulation efficiencies.

Comparing the findings of the heterogeneous binding assay with the homogeneous assay, it is obvious that more information can be gained from the latter. While the heterogeneous assay relies on an immobilized antibody to function well (e.g., clone RM105 adsorbed vs. secondary antibody), the homogeneous assay allows for natural binding events and simplifies the assay procedure. In addition, it provides information on the complement-triggering ability of the antibodies studied. For example, clone RM105 was found to trigger the complement system very well [48]. This aligns well with our results, as clone RM105 had an EC_50_ value more than four times lower than that of clone 6.3 or the anti-biotin antibody (Fig. 3a–c), whereas it performed poorly in the heterogeneous binding assay compared to the other antibodies (Fig. S4). Thus, we postulate that our assay can provide such functional information in a simple assay format. The complement-triggering capability of antibodies generally depends on clonality, isotype, subclass, hinge flexibility, and affinity toward the antigen [49, 50]. In particular, glycosylation of the antibody’s Fc region is essential for C1 complex binding and thus further complement activation [51]. This theoretically allows tuning of complement-triggering capabilities by glyco-engineering of the antibodies [52] and hence adaptation for specific therapeutic applications.

To demonstrate the capabilities of this assay, combinations of antibodies (anti-biotin, anti-PEG clone 6.3 and clone RM105) were investigated using liposomes with both a biotin and methoxy-PEG moiety. Antibody concentrations in the dynamic range were specifically selected for this purpose (Fig. 3d and Fig. S11). It was found that not only did the complement-triggering capabilities of the individual antibodies add up, but that their joint use also led to an enhancing effect in complement-mediated lysis. This is well known in the complement field, where the use of two or more antibodies targeting either different antigens on the membrane surface or different epitopes of an antigen facilitates a synergistic effect and promotes complement activation [53]. Here, maximum liposome lysis (166%) was obtained when all three antibodies were applied. This observation also suggests that two or more antibodies, that do not trigger complement lysis on their own or are present at too low concentrations, might be able to trigger complement lysis when used in combination.

To obtain the traditional antigen-binding ability of an antibody in the homogeneous assay format, the readout must be decoupled from its complement-triggering activity. This was achieved by using a secondary antibody that binds to the antigen-specific antibody, thereby taking over the major role in complement activation. This was demonstrated for the goat anti-biotin antibody (Fig. S12) using a secondary donkey anti-goat antibody. Furthermore, by using saturating concentrations of the secondary antibody, complement activation and hence liposome lysis and signal readout can be maximized. Finally, this also demonstrates that the liposome platform technology is applicable to analytes other than antibodies and can be used for competitive and sandwich assay strategies alike. This will be further explored in future studies.

### Detection of naturally occurring anti-PEG antibodies in human sera

PEG is widely used in various industrial products, particularly in cosmetics and pharmaceutical drugs [54]. In cosmetics, for instance, PEG is applied as a surfactant, cleansing agent, emulsifier, or skin conditioner [55], while PEGylation is a common and popular conjugation strategy in drug delivery, since it decreases renal, proteolytic, and phagocytotic clearance, resulting in higher circulation times and a reduction of adverse effects [54, 56, 57]. Therefore, numerous FDA-approved PEGylated drugs are currently on the market. Not surprisingly, frequent exposure to PEGylated materials triggers the human immune system to produce anti-PEG antibodies. While free PEG has no or a weak immunogenic effect, it can trigger an immune response when conjugated to macromolecules or nanoparticles [54, 58, 59]. The induced antibodies are typically directed against specific motifs of PEG, such as the backbone or terminal groups [47].

**Fig. 4:**
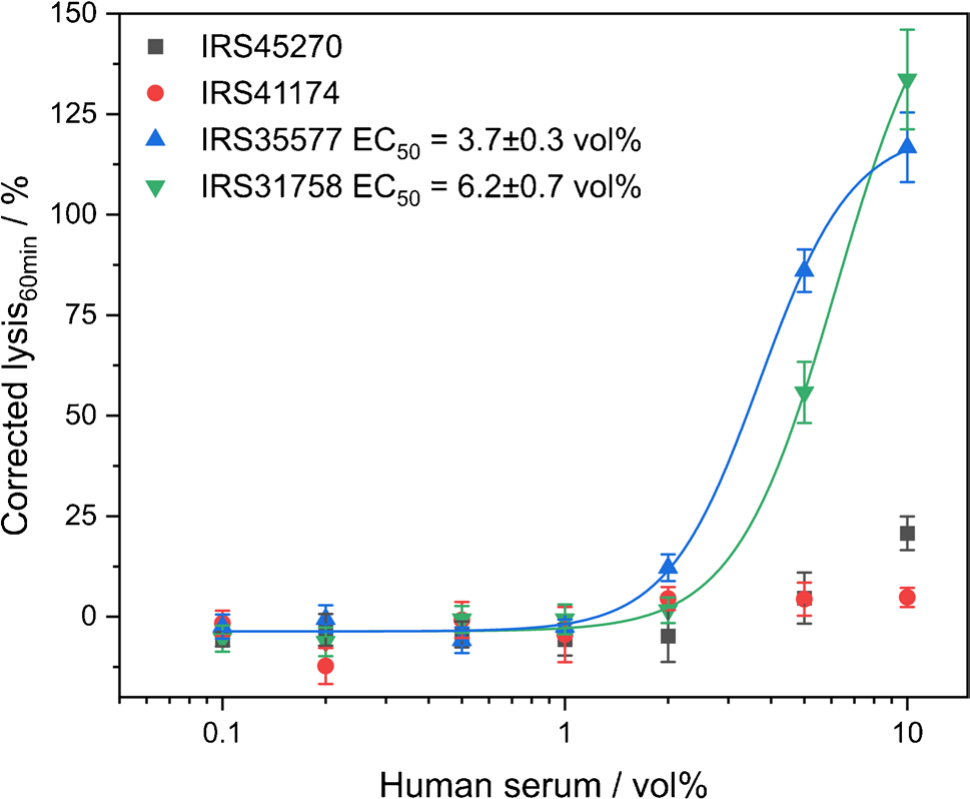
Screening of anti-PEG antibodies in human sera. Corrected lysis values of PEGylated 10 mM SRB-liposomes (1 µM total lipids; batch S1) in a homogeneous complement assay performing a serum titration of different commercial sera (IRS batches 31,758, 35,577, 41,174, and 45,270). 0.1–10 vol% of human sera were used. Fluorescence intensities were adjusted by the iaS signal and normalized to the endpoint fluorescence of the positive control. λ_Ex_ = 565(8) nm and λ_Em_ = 585(8) nm; gain 150. T = 37 °C. n = 3

The natural presence of these complement-active antibodies in human serum samples makes them an ideal model analyte for further optimizing the homogenous liposome-based complement assay and for demonstrating its application potential. Initially, we focused on the overall detection of such antibodies to show the capability of this novel diagnostic platform. Here, the stealthiness of liposomes in dependency on the human serum concentration was investigated using biotinylated liposomes, as no natural anti-biotin antibodies were present in human serum samples. Liposomes remained stealth over a broad range of serum to liposome ratios, whereas increasing serum concentrations—and thus the amount of available complement proteins—led to enhanced antibody-triggered lysis (Fig. S13). This shows that the liposome stealthiness or triggerability does not only depend on the amount of trigger but can also be tuned by the liposomes to serum ratio. Similar observations were made for PEGylated liposomes, which remained stealth when 1 µM total lipids were used in 1 vol% human serum but were lysed by naturally occurring anti-PEG antibodies in 10 vol% serum (Fig. 4). Unlike the approach using biotinylated liposomes, where the trigger amount was kept constant, the quantity of trigger antibodies plays a major role in this case. Here, we also demonstrated that the liposomes remained stealth when both the amount of serum (10 vol%) and liposomes (10 µM total lipids) were increased (Fig. S14) equally, maintaining a constant liposome to serum ratio. This further proves that the liposomes to serum ratio is key for successful complement activation. Eventually, the presence of naturally occurring anti-PEG antibodies in different human sera could be detected using PEGylated liposomes (Fig. 4). EC_50_ values were obtained for two of the pooled commercial sera (IRS35577, IRS31758), one had none present (IRS41174) and one (IRS45270) would require more in-depth analysis due to a slight increase in lysis at high serum concentrations. The EC_50_ values provide insight into the amount of complement-active anti-PEG antibodies in the sera, with lower values indicating a higher presence of these antibodies. As expected, non-PEGylated liposomes were shown to be stealth in active serum under the same conditions, demonstrating that the observed complement lysis originated from naturally occurring anti-PEG antibodies, which only bind to PEGylated liposomes (Fig. S15). Considering that in recent years the scientific community has intensively discussed the occurrence of anti-PEG antibodies in the population as a result of the SARS-CoV- 2 mRNA-based vaccinations, functional, simple assays such as the liposome platform technology are timely developments. Scientists have found that vaccinations with PEGylated lipid nanoparticles boosted the overall anti-PEG antibody levels in individuals. Ju et al. studied plasma from 130 adults vaccinated with either BNT162b2 (Pfizer-BioNTech) or mRNA- 1273 (Moderna) and found that anti-PEG IgG levels increased by a mean of 13.1-fold or 1.78-fold, respectively [60]. Anti-PEG IgM levels were boosted 68.5-fold or 2.64-fold following mRNA- 1273 and BNT162b2 vaccination, respectively [60]. However, the clinical relevance of anti-PEG antibodies has not yet been fully researched. We therefore suggest that the functionality of the liposome platform technology, which not only detects the presence but also the complement-triggering capability of the antibodies, will be very useful.

### Translation of the high-throughput assay to the POC

Considering that the readout result of the liposome platform technology is solely based on the separation of intact from lysed liposomes, a simple detection system can easily be envisioned through a standard LFA. Here, we demonstrate with a proof-of-principle assay that such separation can be accomplished with a visual readout of the SRB-entrapping liposomes. Liposomes containing 44 mol% cholesterol were biotinylated. The high concentration of cholesterol ensured complement activation, whereas biotin enabled capture on a streptavidin test line. Using the same negative and positive controls as in the MTP assay, the expected results were obtained. Intact liposomes provided a strong positive test line signal, whereas lysed liposomes were not captured and hence showed no test line signal (Fig. S16) Such a simple readout strategy may be a valuable tool in the future for companion diagnostics in therapies, e.g., for quick and simple monitoring of the occurrence of anti-PEG antibodies.

### Long-term storage stability

In a long-term storage stability study, the integrity and triggerability or stealthiness of SRB-encapsulating liposomes was monitored at 4 °C, RT, and 37 °C for up to 40 months. The three most relevant types of liposomes were investigated, including low-cholesterol (5 mol%) PEGylated liposomes at a liposome to serum ratio ensuring stealthiness, i.e., 10 µM total lipid with 10 vol% serum, carboxyl-functionalized liposomes, and high-cholesterol (44 mol%) liposomes. Low-cholesterol liposomes were expected to be stealthy, while high-cholesterol liposomes were expected to show lysis throughout the entire study.

**Fig. 5:**
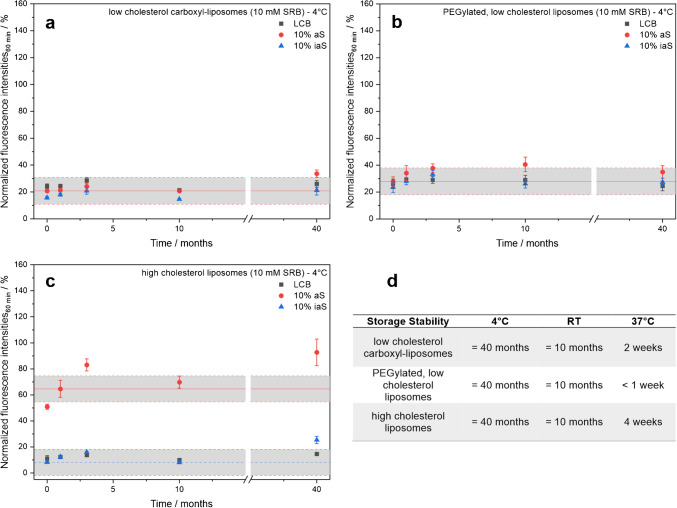
Long-term storage stability study of 10 mM SRB-liposomes. Normalized fluorescence intensities of SRB-liposomes (10 µM total lipids) with different lipid compositions: **a** low-cholesterol, carboxylated (batch S13); **b** low-cholesterol, PEGylated (batch S1); and **c** high-cholesterol, biotinylated (batch S6) in LCB, aS, or iaS throughout the long-term storage stability study up to 40 months at 4 °C. 10 vol% human serum was used as a complement source (PHS for 0–10 months and IRS45270 for the 40 months datapoint). Fluorescence intensities were normalized to the endpoint fluorescence of the positive control. λ_Ex_ = 565(5) nm and λ_Em_ = 585(5) nm; gain 150. T = 37 °C. n = 3. **d** Summary of the liposome storage stability at 4 °C, RT, and 37 °C

It could be shown that storage in buffer at 4 °C ensured colloidal stability and lipid bilayer integrity over the entire study period of 40 months (Fig. 5 and Fig. S17). Interestingly, the last data point (40 months) stands out due to the use of a different pooled serum that probably contained higher concentrations of complement proteins. This may have led to non-stealth behavior of low-cholesterol carboxyl-liposomes and enhanced complement lysis in the case of high-cholesterol liposomes. The latter also showed an increased background signal in inactive serum, which was most likely caused by agglomeration of the liposomes with serum proteins and hence scattering. This suggests that the ratio between total lipids and serum concentration has to be adjusted to avoid non-specific complement lysis, which can also be seen at the highest serum concentration used for biotinylated liposomes (Fig. S13) and newly synthesized carboxyl-liposomes (Fig. S18), ruling out the possibility that it is due to an aging effect of the investigated liposomes, but rather an effect of the serum source.

Liposomes stored at RT were stable for at least 10 months (Figs. S19 and S20). After 15 months, all liposomes revealed substantial lysis in the negative controls. Interestingly, the liposomes showed an amazing stability when stored at 37 °C (Figs. S21 and S22). High-cholesterol liposomes were stable for up to 4 weeks, while low-cholesterol carboxyl-liposomes were stable for 2 weeks. Surprisingly, however, PEGylated liposomes showed unintentional lysis already after 1 week of storage at 37 °C. These data demonstrate not only excellent long-term storage capability of the liposomes, but also a suitability for long-duration assays considering their exceptional stability at 37 °C.

## Conclusion

The developed liposome-based technology provides a novel and versatile homogeneous immunoassay platform for the detection of various analytes in human serum using fluorescent, chemiluminescent, or electrochemical readout strategies. While it was designed for use in an MTP-based HTS, its application in an LFA-based POC format is also feasible. It was found that the serum stability (stealthiness) of the liposomes can be tuned by variation of the cholesterol content. The concept of antibody-triggered complement-induced liposome lysis was shown for selected trigger moieties (PEG, biotin and ARMS peptide) and liposomes with different marker molecules. The main factors influencing the stealthiness and triggerability of liposomes in serum were identified to be the ratio and number of liposomes, trigger molecules, and complement proteins, i.e., the serum content, as well as the total complement activity of the serum. We were able to demonstrate control over these parameters and used them to tune the system toward performance. This platform technology was then successfully applied to detect anti-PEG antibodies using PEGylated liposomes in human sera. This model application suggests that the liposome strategy can be developed into a screening tool for patient and commercial sera to quantify and characterize antibodies in diagnostics and therapeutics, but also to identify specific antibodies that may interfere with certain immunoassay detection strategies. Furthermore, a particularly promising application will be the study of the complement system, especially the interactions of complement proteins and thus the mechanisms of complement activation and regulation. The complement system plays a major role in several diseases, such as systemic lupus erythematosus [61], age-related macular degeneration [62], and atypical hemolytic uremic syndrome [63], but many questions remain. Understanding the underlying mechanisms requires standardizable, routine, and simple assay formats, which the liposome platform can provide. More research is needed, especially to enable control over the separation between patient serum content and inherent complement activity, but commercially available pooled sera will provide a solution. Since the development of such homogeneous platform technologies as presented here will simplify and speed up immunoassay procedures, they are of great importance and form the basis for improving future research and diagnostic methods.

## Supplementary Information

Below is the link to the electronic supplementary material. 

ESM1 (DOCX 2.25 MB)  

## Data Availability

All data supporting the findings of this study are available within the paper and its Electronic Supporting Material.

## References

[1] Psychogios N, Hau DD, Peng J, Guo AC, Mandal R, Bouatra S, Sinelnikov I, Krishnamurthy R, Eisner R, Gautam B, Young N, Xia J, Knox C, Dong E, Huang P, Hollander Z, Pedersen TL, Smith SR, Bamforth F, Greiner R, McManus B, Newman JW, Goodfriend T, Wishart DS. The human serum metabolome. PLoS ONE. 2011;6: e16957. 10.1371/journal.pone.0016957. 21359215 10.1371/journal.pone.0016957 PMC3040193

[2] Plebani M, Banfi G, Bernardini S, Bondanini F, Conti L, Dorizzi R, Ferrara FE, Mancini R, Trenti T. Serum or plasma? An old question looking for new answers. Clin Chem Lab Med. 2020;58:178–87. 10.1515/cclm-2019-0719. 31525152 10.1515/cclm-2019-0719

[3] Carey RN, Jani C, Johnson C, Pearce J, Hui-Ng P, Lacson E. Chemistry Testing on Plasma Versus Serum Samples in Dialysis Patients: Clinical and Quality Improvement Implications. Clin J Am Soc Nephrol. 2016;11:1675–9. 10.2215/CJN.09310915. 27185523 10.2215/CJN.09310915 PMC5012485

[4] Datta P. Immunoassay design. In: Sepulveda JL, Dasgupta A, editors. Accurate Results in the Clinical Laboratory: A Guide to Error Detection and Correction: Elsevier; 2019. p. 69–73. 10.1016/B978-0-12-813776-5.00006-6.

[5] Smith DS, Eremin SA. Fluorescence polarization immunoassays and related methods for simple, high-throughput screening of small molecules. Anal Bioanal Chem. 2008;391:1499–507. 10.1007/s00216-008-1897-z. 18264817 10.1007/s00216-008-1897-z

[6] Padoan A, Cosma C, Sciacovelli L, Faggian D, Plebani M. Analytical performances of a chemiluminescence immunoassay for SARS-CoV-2 IgM/IgG and antibody kinetics. Clin Chem Lab Med. 2020;58:1081–8. 10.1515/cclm-2020-0443. 32301749 10.1515/cclm-2020-0443

[7] Price CP, Trull AK, Berry D, Gorman EG. Development and validation of a particle-enhanced turbidimetric immunoassay for C-reactive protein. J Immunol Methods. 1987;99:205–11. 10.1016/0022-1759(87)90129-3. 3584992 10.1016/0022-1759(87)90129-3

[8] Herget-Rosenthal S, Feldkamp T, Volbracht L, Kribben A. Measurement of urinary cystatin C by particle-enhanced nephelometric immunoassay: precision, interferences, stability and reference range. Ann Clin Biochem. 2004;41:111–8. 10.1258/000456304322879980. 15025800 10.1258/000456304322879980

[9] Lewellen LJ, McCurdy HH. A novel procedure for the analysis of drugs in whole blood by homogeneous enzyme immunoassay (EMIT). J Anal Toxicol. 1988;12:260–4. 10.1093/jat/12.5.260. 2906382 10.1093/jat/12.5.260

[10] Jeon SI, Yang X, Andrade JD. Modeling of homogeneous cloned enzyme donor immunoassay. Anal Biochem. 2004;333:136–47. 10.1016/j.ab.2004.06.044. 15351290 10.1016/j.ab.2004.06.044

[11] Bangham AD, Standish MM, Watkins JC. Diffusion of univalent ions across the lamellae of swollen phospholipids. J Mol Biol. 1965;13:238–52. 10.1016/s0022-2836(65)80093-6. 5859039 10.1016/s0022-2836(65)80093-6

[12] Streif S, Neckermann P, Spitzenberg C, Weiss K, Hoecherl K, Kulikowski K, Hahner S, Noelting C, Einhauser S, Peterhoff D, Asam C, Wagner R, Baeumner AJ. Liposome-based high-throughput and point-of-care assays toward the quick, simple, and sensitive detection of neutralizing antibodies against SARS-CoV-2 in patient sera. Anal Bioanal Chem. 2023;415:1421–35. 10.1007/s00216-023-04548-3. 36754874 10.1007/s00216-023-04548-3 PMC9909147

[13] Torchilin V. Antibody-modified liposomes for cancer chemotherapy. Expert Opin Drug Deliv. 2008;5:1003–25. 10.1517/17425247.5.9.1003. 18754750 10.1517/17425247.5.9.1003

[14] Gerstl F, Loessl M, Borggraefe V, Baeumner AJ. Multiplexed electrochemical liposomes applied to the detection of nucleic acids for Influenza A, Influenza B and SARS-CoV-2. Anal Bioanal Chem. 2024;416:3487–500. 10.1007/s00216-024-05145-8. 38240795 10.1007/s00216-024-05145-8 PMC11156727

[15] Mayer M, Takegami S, Neumeier M, Rink S, Jacobi von Wangelin A, Schulte S, Vollmer M, Griesbeck AG, Duerkop A, Baeumner AJ. Electrochemiluminescence Bioassays with a Water-Soluble Luminol Derivative Can Outperform Fluorescence Assays. Angew Chem Int Ed Engl. 2018;57:408–11. 10.1002/anie.201708630. 10.1002/anie.201708630 29119667

[16] Song S, Liu D, Peng J, Sun Y, Li Z, Gu J-R, Xu Y. Peptide ligand-mediated liposome distribution and targeting to EGFR expressing tumor in vivo. Int J Pharm. 2008;363:155–61. 10.1016/j.ijpharm.2008.07.012. 18692120 10.1016/j.ijpharm.2008.07.012

[17] de Lima PHC, Butera AP, Cabeça LF, Ribeiro-Viana RM. Liposome surface modification by phospholipid chemical reactions. Chem Phys Lipid. 2021;237: 105084. 10.1016/j.chemphyslip.2021.105084. 10.1016/j.chemphyslip.2021.105084 33891960

[18] Barenholz Y. Doxil®–the first FDA-approved nano-drug: lessons learned. J Control Release. 2012;160:117–34. 10.1016/j.jconrel.2012.03.020. 22484195 10.1016/j.jconrel.2012.03.020

[19] Marsanasco M, Márquez AL, Wagner JR, Del V. Alonso S, Chiaramoni NS. Liposomes as vehicles for vitamins E and C: An alternative to fortify orange juice and offer vitamin C protection after heat treatment. Food Research International. 2011;44:3039–46. 10.1016/j.foodres.2011.07.025.

[20] Karny A, Zinger A, Kajal A, Shainsky-Roitman J, Schroeder A. Therapeutic nanoparticles penetrate leaves and deliver nutrients to agricultural crops. Sci Rep. 2018;8:7589. 10.1038/s41598-018-25197-y. 29773873 10.1038/s41598-018-25197-y PMC5958142

[21] Rahimpour Y, Hamishehkar H. Liposomes in cosmeceutics. Expert Opin Drug Deliv. 2012;9:443–55. 10.1517/17425247.2012.666968. 22413847 10.1517/17425247.2012.666968

[22] Mozafari MR. Nanoliposomes: preparation and analysis. Methods Mol Biol. 2010;605:29–50. 10.1007/978-1-60327-360-2_2. 20072871 10.1007/978-1-60327-360-2_2

[23] Tenchov R, Bird R, Curtze AE, Zhou Q. Lipid Nanoparticles─From Liposomes to mRNA Vaccine Delivery, a Landscape of Research Diversity and Advancement. ACS Nano. 2021;15:16982–7015. 10.1021/acsnano.1c04996. 34181394 10.1021/acsnano.1c04996

[24] Vamvakaki V, Chaniotakis NA. Pesticide detection with a liposome-based nano-biosensor. Biosens Bioelectron. 2007;22:2848–53. 10.1016/j.bios.2006.11.024. 17223333 10.1016/j.bios.2006.11.024

[25] Cunningham CM, Kingzette M, Richards RL, Alving CR, Lint TF, Gewurz H. Activation of Human Complement by Liposomes: A Model for Membrane Activation of the Alternative Pathway. J Immunol. 1979;122:1237–42. 10.4049/jimmunol.122.4.1237. 448089

[26] Richards RL, Gewurz H, Osmand AP, Alving CR. Interactions of C-reactive protein and complement with liposomes. Proc Natl Acad Sci U S A. 1977;74:5672–6. 10.1073/pnas.74.12.5672. 271994 10.1073/pnas.74.12.5672 PMC431855

[27] Kishimura M, Yamaji H, Fukuda H, Terashima M, Katoh S, Sada E. A simple method for measuring the complement activities of both classical and alternative pathways by using rabbit γ-globulin-coupled liposomes. J Ferment Bioeng. 1989;68:395–8. 10.1016/0922-338X(89)90093-7.

[28] Jaskowski TD, Martins TB, Litwin CM, Hill HR. Comparison of three different methods for measuring classical pathway complement activity. Clin Diagn Lab Immunol. 1999;6:137–9. 10.1128/CDLI.6.1.137-139.1999. 9874678 10.1128/cdli.6.1.137-139.1999 PMC95674

[29] Ghebrehiwet B. The complement system: an evolution in progress. F1000Res. 2016;5:2840. 10.12688/f1000research.10065.1. 10.12688/f1000research.10065.1 PMC5155499 27990282

[30] Merle NS, Church SE, Fremeaux-Bacchi V, Roumenina LT. Complement System Part I - Molecular Mechanisms of Activation and Regulation. Front Immunol. 2015;6:262. 10.3389/fimmu.2015.00262. 26082779 10.3389/fimmu.2015.00262 PMC4451739

[31] Beurskens FJ, van Schaarenburg RA, Trouw LA. C1q, antibodies and anti-C1q autoantibodies. Mol Immunol. 2015;68:6–13. 10.1016/j.molimm.2015.05.010. 26032012 10.1016/j.molimm.2015.05.010

[32] Bubeck D. The making of a macromolecular machine: assembly of the membrane attack complex. Biochemistry. 2014;53:1908–15. 10.1021/bi500157z. 24597946 10.1021/bi500157z

[33] Schäfer N, Grosche A, Reinders J, Hauck SM, Pouw RB, Kuijpers TW, Wouters D, Ehrenstein B, Enzmann V, Zipfel PF, Skerka C, Pauly D. Complement Regulator FHR-3 Is Elevated either Locally or Systemically in a Selection of Autoimmune Diseases. Front Immunol. 2016;7:542. 10.3389/fimmu.2016.00542. 27965669 10.3389/fimmu.2016.00542 PMC5124756

[34] Edwards KA, Curtis KL, Sailor JL, Baeumner AJ. Universal liposomes: preparation and usage for the detection of mRNA. Anal Bioanal Chem. 2008;391:1689–702. 10.1007/s00216-008-1992-1. 18327569 10.1007/s00216-008-1992-1

[35] Schneider CA, Rasband WS, Eliceiri KW. NIH Image to ImageJ: 25 years of image analysis. Nat Methods. 2012;9:671–5. 10.1038/nmeth.2089. 22930834 10.1038/nmeth.2089 PMC5554542

[36] Redondo-Morata L, Giannotti MI, Sanz F. Influence of cholesterol on the phase transition of lipid bilayers: a temperature-controlled force spectroscopy study. Langmuir. 2012;28:12851–60. 10.1021/la302620t. 22873775 10.1021/la302620t

[37] Moein Moghimi S, Hamad I, Bünger R, Andresen TL, Jørgensen K, Hunter AC, Baranji L, Rosivall L, Szebeni J. Activation of the human complement system by cholesterol-rich and PEGylated liposomes-modulation of cholesterol-rich liposome-mediated complement activation by elevated serum LDL and HDL levels. J Liposome Res. 2006;16:167–74. 10.1080/08982100600848801. 16952871 10.1080/08982100600848801

[38] Niyonzima N, Halvorsen B, Sporsheim B, Garred P, Aukrust P, Mollnes TE, Espevik T. Complement activation by cholesterol crystals triggers a subsequent cytokine response. Mol Immunol. 2017;84:43–50. 10.1016/j.molimm.2016.09.019. 27692470 10.1016/j.molimm.2016.09.019

[39] Devine DV, Wong K, Serrano K, Chonn A, Cullis PR. Liposome-complement interactions in rat serum: implications for liposome survival studies. Biochim Biophys Acta. 1994;1191:43–51. 10.1016/0005-2736(94)90231-3. 8155683 10.1016/0005-2736(94)90231-3

[40] Alving CR, Richards RL, Guirguis AA. Cholesterol-dependent human complement activation resulting in damage to liposomal model membranes. J Immunol. 1977;118:342–7. 830757

[41] Hughes LD, Rawle RJ, Boxer SG. Choose your label wisely: water-soluble fluorophores often interact with lipid bilayers. PLoS ONE. 2014;9: e87649. 10.1371/journal.pone.0087649. 24503716 10.1371/journal.pone.0087649 PMC3913624

[42] Micklisch S, Lin Y, Jacob S, Karlstetter M, Dannhausen K, Dasari P, von der Heide M, Dahse H-M, Schmölz L, Grassmann F, Alene M, Fauser S, Neumann H, Lorkowski S, Pauly D, Weber BH, Joussen AM, Langmann T, Zipfel PF, Skerka C. Age-related macular degeneration associated polymorphism rs10490924 in ARMS2 results in deficiency of a complement activator. J Neuroinflammation. 2017;14:4. 10.1186/s12974-016-0776-3. 28086806 10.1186/s12974-016-0776-3 PMC5234120

[43] Onishchenko N, Tretiakova D, Vodovozova E. Spotlight on the protein corona of liposomes. Acta Biomater. 2021;134:57–78. 10.1016/j.actbio.2021.07.074. 34364016 10.1016/j.actbio.2021.07.074

[44] Ritz S, Schöttler S, Kotman N, Baier G, Musyanovych A, Kuharev J, Landfester K, Schild H, Jahn O, Tenzer S, Mailänder V. Protein corona of nanoparticles: distinct proteins regulate the cellular uptake. Biomacromol. 2015;16:1311–21. 10.1021/acs.biomac.5b00108. 10.1021/acs.biomac.5b00108 25794196

[45] Partikel K, Korte R, Stein NC, Mulac D, Herrmann FC, Humpf H-U, Langer K. Effect of nanoparticle size and PEGylation on the protein corona of PLGA nanoparticles. Eur J Pharm Biopharm. 2019;141:70–80. 10.1016/j.ejpb.2019.05.006. 31082511 10.1016/j.ejpb.2019.05.006

[46] Kitamura M, Murakami K, Yamada K, Kawai K, Kunishima M. Binding of sulforhodamine B to human serum albumin: A spectroscopic study. Dyes Pigm. 2013;99:588–93. 10.1016/j.dyepig.2013.06.011.

[47] Saifer MGP, Williams LD, Sobczyk MA, Michaels SJ, Sherman MR. Selectivity of binding of PEGs and PEG-like oligomers to anti-PEG antibodies induced by methoxyPEG-proteins. Mol Immunol. 2014;57:236–46. 10.1016/j.molimm.2013.07.014. 24200843 10.1016/j.molimm.2013.07.014

[48] Estapé Senti M, de Jongh CA, Dijkxhoorn K, Verhoef JJF, Szebeni J, Storm G, Hack CE, Schiffelers RM, Fens MH, Boross P. Anti-PEG antibodies compromise the integrity of PEGylated lipid-based nanoparticles via complement. J Control Release. 2022;341:475–86. 10.1016/j.jconrel.2021.11.042. 34890719 10.1016/j.jconrel.2021.11.042

[49] Goldberg BS, Ackerman ME. Antibody-mediated complement activation in pathology and protection. Immunol Cell Biol. 2020;98:305–17. 10.1111/imcb.12324. 32142167 10.1111/imcb.12324 PMC7293394

[50] Diebolder CA, Beurskens FJ, de Jong RN, Koning RI, Strumane K, Lindorfer MA, Voorhorst M, Ugurlar D, Rosati S, Heck AJR, van de Winkel JGJ, Wilson IA, Koster AJ, Taylor RP, Saphire EO, Burton DR, Schuurman J, Gros P, Parren PWHI. Complement is activated by IgG hexamers assembled at the cell surface. Science. 2014;343:1260–3. 10.1126/science.1248943. 24626930 10.1126/science.1248943 PMC4250092

[51] Jefferis R. Isotype and glycoform selection for antibody therapeutics. Arch Biochem Biophys. 2012;526:159–66. 10.1016/j.abb.2012.03.021. 22465822 10.1016/j.abb.2012.03.021

[52] Natsume A, In M, Takamura H, Nakagawa T, Shimizu Y, Kitajima K, Wakitani M, Ohta S, Satoh M, Shitara K, Niwa R. Engineered antibodies of IgG1/IgG3 mixed isotype with enhanced cytotoxic activities. Cancer Res. 2008;68:3863–72. 10.1158/0008-5472.CAN-07-6297. 18483271 10.1158/0008-5472.CAN-07-6297

[53] Rijkers M, Schmidt D, Lu N, Kramer CSM, Heidt S, Mulder A, Porcelijn L, Claas FHJ, Leebeek FWG, Jansen AJG, Jongerius I, Zeerleder SS, Vidarsson G, Voorberg J, de Haas M. Anti-HLA antibodies with complementary and synergistic interaction geometries promote classical complement activation on platelets. Haematologica. 2019;104:403–16. 10.3324/haematol.2018.201665. 30262558 10.3324/haematol.2018.201665 PMC6355480

[54] Kozma GT, Shimizu T, Ishida T, Szebeni J. Anti-PEG antibodies: Properties, formation, testing and role in adverse immune reactions to PEGylated nano-biopharmaceuticals. Adv Drug Deliv Rev. 2020;154–155:163–75. 10.1016/j.addr.2020.07.024. 32745496 10.1016/j.addr.2020.07.024

[55] Jang H-J, Shin CY, Kim K-B. Safety Evaluation of Polyethylene Glycol (PEG) Compounds for Cosmetic Use. Toxicol Res. 2015;31:105–36. 10.5487/TR.2015.31.2.105. 26191379 10.5487/TR.2015.31.2.105 PMC4505343

[56] Armstrong JK. The occurrence, induction, specificity and potential effect of antibodies against poly(ethylene glycol). In: Veronese FM, editor. Regylated protein drugs: Basic science and clinical applications. Boston: Birkhauser; 2009. p. 147–168. 10.1007/978-3-7643-8679-5_9.

[57] Zhang P, Sun F, Liu S, Jiang S. Anti-PEG antibodies in the clinic: Current issues and beyond PEGylation. J Control Release. 2016;244:184–93. 10.1016/j.jconrel.2016.06.040. 27369864 10.1016/j.jconrel.2016.06.040 PMC5747248

[58] Richter AW, Akerblom E. Antibodies against polyethylene glycol produced in animals by immunization with monomethoxy polyethylene glycol modified proteins. Int Arch Allergy Appl Immunol. 1983;70:124–31. 10.1159/000233309. 6401699 10.1159/000233309

[59] Richter AW, Akerblom E. Polyethylene glycol reactive antibodies in man: titer distribution in allergic patients treated with monomethoxy polyethylene glycol modified allergens or placebo, and in healthy blood donors. Int Arch Allergy Appl Immunol. 1984;74:36–9. 10.1159/000233512. 6706424 10.1159/000233512

[60] Ju Y, Lee WS, Pilkington EH, Kelly HG, Li S, Selva KJ, Wragg KM, Subbarao K, Nguyen THO, Rowntree LC, Allen LF, Bond K, Williamson DA, Truong NP, Plebanski M, Kedzierska K, Mahanty S, Chung AW, Caruso F, Wheatley AK, Juno JA, Kent SJ. Anti-PEG Antibodies Boosted in Humans by SARS-CoV-2 Lipid Nanoparticle mRNA Vaccine. ACS Nano. 2022;16:11769–80. 10.1021/acsnano.2c04543. 35758934 10.1021/acsnano.2c04543

[61] Pettigrew HD, Teuber SS, Gershwin ME. Clinical Significance of Complement Deficiencies. Ann N Y Acad Sci. 2009;1173:108–23. 10.1111/j.1749-6632.2009.04633.x. 19758139 10.1111/j.1749-6632.2009.04633.x

[62] Klein RJ, Zeiss C, Chew EY, Tsai J-Y, Sackler RS, Haynes C, Henning AK, SanGiovanni JP, Mane SM, Mayne ST, Bracken MB, Ferris FL, Ott J, Barnstable C, Hoh J. Complement factor H polymorphism in age-related macular degeneration. Science. 2005;308:385–9. 10.1126/science.1109557. 15761122 10.1126/science.1109557 PMC1512523

[63] Noris M, Remuzzi G. Atypical hemolytic-uremic syndrome. N Engl J Med. 2009;361:1676–87. 10.1056/NEJMra0902814. 19846853 10.1056/NEJMra0902814

