# Supplementary material for: A homogeneous immunoassay technology based on liposomes and the complement system enables one-step, no-wash, rapid diagnostics directly in serum
Source: Anal Bioanal Chem. 2025 May 2;417(15):3257–73. doi: 10.1007/s00216-025-05882-4 (PMC12122657; doi:10.1007/s00216-025-05882-4)
Supplement: Supplementary file 1 — (DOCX 2.25 MB) [file 216_2025_5882_MOESM1_ESM.docx]

**Electronic Supporting Material**

**A homogeneous immunoassay technology based on liposomes and the complement system enables one-step, no-wash, rapid diagnostics directly in serum**

Kilian Hoecherl^1^, Simon Streif^1^, Clemens Spitzenberg^1^, Simone Rink^1^, Arne Behrent^1^, Ferdinand Holzhausen^1^, Christian Griesche^1^, Cornelia Rogoll^1^, Maximilian Foedlmeier^1^, Anna Gebhard^1^, Kacper Kulikowski^1^, Nicole Schaefer^2^, Diana Pauly^3^, Antje J. Baeumner^1*^

^1^ Institute of Analytical Chemistry, Chemo‑ and Biosensors, University of Regensburg, Universitätsstraße 31, 93053 Regensburg, Germany

^2^ Department of Orthopaedic Surgery, Experimental Orthopaedics, Centre for Medical Biotechnology (ZMB/Biopark 1), University of Regensburg, Universitätsstraße 31, 93053 Regensburg, Germany

^3^ Experimental Ophthalmology, University of Marburg, Baldingerstraße, 35043 Marburg

***Author for correspondence:**

**Email: antje.baeumner@ur.de**

**Phone: +49 941 943 4065**

**Postal address:**

**Antje Baeumner**

**Universität Regensburg**

**93040 Regensburg**

**Germany**

1. **Experimental section**
   1. **Chemicals and consumables**

Sucrose, sodium azide, sodium chloride, potassium hydrogen carbonate and dialysis membrane Spectra/Por© 4 (MWCO: 12-14 kDa) (2718.1) were purchased from Carl Roth. Phosphorous standard was obtained from Bernd Kraft GmbH (Germany). Chloroform, methanol and Spectra-Por® Float-A-Lyzer® G2 (1 mL, MWCO: 1000 kDa) were purchased from Fisher Scientific. Tetrasodium ethylenediaminetetraacetic acid (EDTA), ethylene glycol-bis (β-aminoethyl ether)-N,N,N’,N’-tetraacetic acid (EGTA), disodium hydrogen phosphate dihydrate, potassium dihydrogen phosphate, glycine, potassium chloride, Whatman Nucleopore™ Track-Etched membranes (1.0 µm, 0.4 µm and 0.2 µm diameter) and Tween® 20 were obtained from Sigma Aldrich/Merck (Germany). Polyvinylpyrrolidone was purchased from Serva Electrophoresis GmbH (Germany).

- 1. **Buffer compositions**

**Table S1** Buffer compositions

| Buffer | Composition |
| --- | --- |
| HEPES-saline-sucrose buffer (HSS) pH 7.5 | 10 mM HEPES  200 mM NaCl  200 mM sucrose  0.01 w/v% NaN_3_ |
| Phosphate-buffered saline (PBS) pH 7.4 | 137 mM NaCl  2.7 mM KCl  10 mM Na_2_HPO_4_  1.8 mM KH_2_PO_4_ |
| PBS-T | 0.1 w% Tween 20 in PBS |
| Liposome complement buffer (LCB) | 10 mM HEPES  150 mM NaCl  135 nM CaCl_2_  1 mM MgCl_2_ |
| MES buffer pH 6.0 | 0.1 M MES |
| Inactivation complement buffer (iaCB)  pH~8 | 200 mM EDTA 0.5 µM EGTA in LCB |
| Glycine-NaOH buffer  pH 8.6 | 10 mM glycine  200 mM NaCl  100 mM sucrose  0.01 w/v% NaN_3_ |
| Carbonate-buffer saline (CBS)  pH 10.5 | 10 mM KHCO_3_  200 mM NaCl  100 mM sucrose |
| LFA running buffer | LCB with 0.4 w% PVP  0.1 w% Tween 20  200 mM CaCl_2_ |
| LFA washing buffer | LCB with 0.2 w% PVP  0.05 w% Tween 20  100 mM CaCl_2_ |

1. **Results**

**Table S2** Characteristics of electrochemical liposomes encapsulating 200 mM RuHex.

| batch | Lipid composition | Surface | Hydrodynamic diameter / nm | PDI | Zeta-potential / mV | Total lipid concentration/ mM |
| --- | --- | --- | --- | --- | --- | --- |
| R1 | DPPC: 35 mol%  DPPG: 19 mol%  Cholesterol: 46 mol% | anionic | 260 ± 2 | 0.16 ± 0.01 | -23.4 ± 1.3 | 7.67 ± 0.04 |
| R2 | DPPC: 71 mol%  DPPG: 19 mol%  Cholesterol: 5 mol%  DPPE-biotin: 2 mol%  N-Glutaryl-DPPE: 2 mol% | anionic  biotin  COOH | 187 ± 2 | 0.22 ± 0.01 | -20.1 ± 1.3 | 7.76 ± 0.07 |

**Table S3** Characteristics of 30 mM *m*-carboxy luminol-encapsulating liposomes.

| batch | Lipid composition | Surface | Hydrodynamic diameter / nm | PDI | Zeta-potential / mV | Total lipid concentration/ mM |
| --- | --- | --- | --- | --- | --- | --- |
| CL1 | DPPC: 35 mol%  DPPG: 18 mol%  Cholesterol: 45 mol%  DPPE-biotin: 2 mol% | anionic  biotin | 135 ± 1 | 0.08 ± 0.02 | -28.7 ± 1.6 | 15.27 ± 0.02 |
| CL2 | DPPC: 75 mol%  DPPG: 17 mol%  Cholesterol: 6 mol%  DPPE-biotin: 2 mol% | anionic  biotin | 123 ± 3 | 0.09 ± 0.02 | -19 ± 1 | 9.60 ± 0.04 |

**Table S4** Characteristics of SRB-encapsulating liposomes.

| batch | Lipid composition | Surface | Encapsulant | Hydrodynamic diameter / nm | PDI | Zeta-potential / mV | Total lipid concentration/ mM | Initial fluorescence / % |
| --- | --- | --- | --- | --- | --- | --- | --- | --- |
| S1 | DPPC: 72 mol%  DPPG: 19 mol%  Cholesterol: 5 mol%  DMPE-PEG2k: 2 mol%  DMPE-PEG2k-biotin: 2 mol% | anionic  PEG-biotin | 10 mM SRB  210 mM NaCl | 135 ± 3 | 0.13 ± 0.02 | -3.0 ± 1.7 | 10.40 ± 0.04 | 16.3 ± 1.7 |
| S2 | DPPC: 67 mol%  DPPG: 19 mol%  Cholesterol: 10 mol%  DMPE-PEG2k: 2 mol%  DMPE-PEG2k-biotin: 2 mol% | anionic  PEG-biotin | 10 mM SRB  210 mM NaCl | 128 ± 1 | 0.11 ± 0.01 | -2.0 ± 0.5 | 13.09 ± 0.06 | 28.5 ± 1.9 |
| S3 | DPPC: 62 mol%  DPPG: 19 mol%  Cholesterol: 15 mol%  DMPE-PEG2k: 2 mol%  DMPE-PEG2k-biotin: 2 mol% | anionic  PEG-biotin | 10 mM SRB  210 mM NaCl | 138 ± 2 | 0.18 ± 0.02 | -3.1 ± 0.7 | 10.73 ± 0.10 | 11.3 ± 0.9 |
| S4 | DPPC: 57 mol%  DPPG: 19 mol%  Cholesterol: 20 mol%  DMPE-PEG2k: 2 mol%  DMPE-PEG2k-biotin: 2 mol% | anionic  PEG-biotin | 10 mM SRB  210 mM NaCl | 144 ± 2 | 0.22 ± 0.01 | -1.8 ± 0.6 | 13.45 ± 0.07 | 10.5 ± 1.3 |
| S5 | DPPC: 52 mol%  DPPG: 19 mol%  Cholesterol: 25 mol%  DMPE-PEG2k: 2 mol%  DMPE-PEG2k-biotin: 2 mol% | anionic  PEG-biotin | 10 mM SRB  210 mM NaCl | 148 ± 1 | 0.21 ± 0.01 | -1.5 ± 1.4 | 7.90 ± 0.02 | 9.7 ± 0.5 |
| S6 | DPPC: 34 mol%  DPPG: 20 mol%  Cholesterol: 44 mol%  DPPE-biotin: 2 mol% | anionic  biotin | 10 mM SRB  210 mM NaCl | 272 ± 4 | 0.25 ± 0.01 | -24.4 ± 2.8 | 8.9 ± 0.4 | 5.0 ± 0.5 |
| S7 | DPPC: 47 mol%  DPPG: 19 mol%  Cholesterol: 30 mol%  N-Glutaryl-DPPE: 4 mol% | anionic  COOH | 50 mM SRB  200 mM NaCl | 148 ± 1 | 0.06 ± 0.01 | -27 ± 3 | 11.93 ± 0.07 | 0.4 ± 0.1 |

| batch | Lipid composition | Surface | Encapsulant | Hydrodynamic diameter / nm | PDI | Zeta-potential / mV | Total lipid concentration/ mM | Initial fluorescence / % |
| --- | --- | --- | --- | --- | --- | --- | --- | --- |
| S8 | DPPC: 47 mol%  DPPG: 19 mol%  Cholesterol: 30 mol%  N-Glutaryl-DPPE: 4 mol% | anionic  COOH | 25 mM SRB  200 mM NaCl | 156 ± 9 | 0.08 ± 0.02 | -23.9 ± 0.9 | 10.50 ± 0.02 | 0.9 ± 0.2 |
| S9 | DPPC: 67 mol%  DPPG: 19 mol%  Cholesterol: 10 mol%  N-Glutaryl-DPPE: 4 mol% | anionic  COOH | 10 mM SRB  210 mM NaCl | 124 ± 4 | 0.08 ± 0.01 | -22.6 ± 2.8 | 11.51 ± 0.03 | 12.2 ± 1.0 |
| S10 | DPPC: 72 mol%  DPPG: 19 mol%  Cholesterol: 5 mol%  N-Glutaryl-DPPE: 4 mol% | anionic  COOH | 10 mM SRB  210 mM NaCl | 128 ± 5 | 0.09 ± 0.03 | -17 ± 2 | 6.73 ± 0.02 | 14.1 ± 0.6 |
| S11 | DPPC: 72 mol%  DPPG: 19 mol%  Cholesterol: 5 mol%  N-Glutaryl-DPPE: 4 mol%  Modified with 0.5 mol% ARMS | anionic  COOH  ARMS | 10 mM SRB  210 mM NaCl | 3762 ± 794 | 0.65 ± 0.33 | -21.9 ± 1.1 | 4.07 ± 0.02 | - |
| S12 | DPPC: 73 mol%  DPPG: 20 mol%  Cholesterol: 5 mol%  DPPE-biotin: 2 mol% | anionic  biotin | 10 mM SRB  210 mM NaCl | 104 ± 2 | 0.14 ± 0.01 | -12.1 ± 0.4 | 3.42 ± 0.01 | 9.9 ± 0.8 |
| S13 | DPPC: 72 mol%  DPPG: 19 mol%  Cholesterol: 5 mol%  N-Glutaryl-DPPE: 4 mol% | anionic  COOH | 10 mM SRB  210 mM NaCl | 199 ± 13 | 0.26 ± 0.02 | -22.0 ± 1.6 | 9.25 ± 0.03 | 8.0 ± 0.6 |
| S14 | DPPC: 72 mol%  DPPG: 19 mol%  Cholesterol: 5 mol%  N-Glutaryl-DPPE: 4 mol% | anionic  COOH | 10 mM SRB  210 mM NaCl | 174 ± 1 | 0.20 ± 0.01 | -20.0 ± 1.0 | 5.92 ± 0.02 | 8.9 ± 0.5 |

| batch | Lipid composition | Surface | Encapsulant | Hydrodynamic diameter / nm | PDI | Zeta-potential / mV | Total lipid concentration/ mM | Initial fluorescence / % |
| --- | --- | --- | --- | --- | --- | --- | --- | --- |
| S15 | DPPC: 74 mol%  DPPG: 19 mol%  Cholesterol: 5 mol%  DMPE-PEG2k-biotin: 2 mol% | anionic  PEG-biotin | 10 mM SRB  210 mM NaCl | 184 ± 4 | 0.19 ± 0.03 | -1.4 ± 1.6 | 8.57 ± 0.03 | 11.5 ± 1.0 |
| S16 | DPPC: 34 mol%  DPPG: 19 mol%  Cholesterol: 44 mol%  DPPE-biotin: 2 mol% | anionic  biotin | 10 mM SRB  210 mM NaCl | 422 ± 9 | 0.18 ± 0.01 | -23.6 ± 2.3 | 7.80 ± 0.52 | 5.4 ± 0.6 |
| S17 | DPPC: 73 mol%  DPPG: 18 mol%  Cholesterol: 5 mol%  N-Glutaryl-DPPE: 4 mol% | anionic  COOH | 10 mM SRB  210 mM NaCl | 138 ± 4 | 0.10 ± 0.01 | -21.8 ± 1.2 | 10.49 ± 0.02 | 8.4 ± 0.7 |

**a**

**b**

**Fig. S1** Electrochemical complement assay using RuHex-encapsulating liposomes. Peak heights of **(a)** stealth, 5 mol% cholesterol (batch R2) or **(b)** complement-triggering, 44 mol% cholesterol (batch R1) 200 mM RuHex-liposomes (100 µM total lipids) on a DropSens screen-printed carbon electrode (DRP-110). 10 vol% human serum was used as complement source (IRS33478). Square wave voltammograms were recorded from -0.5 V to 0.1 V. E_step_ = 4 mV, E_amp_ = 40 mV, f = 2 Hz. n = 1.

**Fig. S2** Square wave voltammograms of K_4_[Fe(CN)_6_] vs [Ru(NH_3_)_6_]Cl_3_ in PBS measured on a DropSens screen-printed carbon electrode (DRP-100). Square wave voltammograms were recorded from -0.6 V to 0.5 V. t_quiet_ = 3 s, E_step_ = 5 mV, E_amp_ = 50 mV, f = 5 Hz.

**a**

**b**

**Fig S3** Square wave voltammograms of **(A)** [Ru(NH_3_)_6_]Cl_3_ and **(B)** K_4_[Fe(CN)_6_] in 25 vol% human serum (S1764) in PBS measured on a DropSens screen-printed carbon electrode (DRP-110). t_quiet_ = 3 s, E_step_ = 5 mV, E_amp_ = 50 mV, f = 5 Hz.

**a**

**b**

**Fig. S4** Binding assay of methoxy-PEG and PEG-biotin (batch S1), only PEG-biotin (batch S15) or biotinylated (batch S12) liposomes immobilized (10 µM total lipids in 100 µL HSS) on a Nunc MaxiSorp high binding MTP coated with 2 µg/mL **(a)** anti-PEG/-biotin or **(b)** anti-rabbit/-mouse antibodies. The plate was blocked with 1 w/v% BSA in PBS-T. Secondary antibodies were further incubated with 2 µg/mL anti-PEG antibodies (clone RM105 or 6.3; 1 h at RT and 300 rpm). After washing with PBS or PBS-T (2x, 150 µL) and HSS (3x, 150 µL), liposomes were incubated for 3 h at RT and 300 rpm, washed with HSS (3x, 150 µL) and lysed by addition of 30 mM OG in double dist. H_2_O (100 µL, 10 min inc., RT, 300 rpm). λ_Ex_ = 560(10) nm and λ_Em_ = 585(10) nm, gain 100. n = 3.

**Fig. S5** Screening of various additives to decrease the background signal in the homogeneous complement assay using 10 mM SRB-liposomes (1 µM total lipids; batch S14). Fluorescence measurements were carried out in LCB. λ_Ex_ = 565(8) nm and λ_Em_ = 585(8) nm; gain 150. n = 3.

**a**

**b**

**Fig. S6** Investigation of the influence of serum and OG on the fluorescence of SRB and SRB-liposomes. Time-resolved fluorescence intensities of **(a)** 1.5 µM SRB or **(b)** 10 µM anionic low-cholesterol liposomes (10 µM total lipids; batch S12) in 30 mM OG with (IRS41174) or without human serum. As a lysed liposome control, liposomes were diluted to 100 µM in double dist. H_2_O and ultrasonicated for >30 min. Experiments were performed in LCB. Serum source was IR41174. λ_Ex_ = 565(8) nm and λ_Em_ =585(8) nm; gain 125. T = 37°C. n = 3.


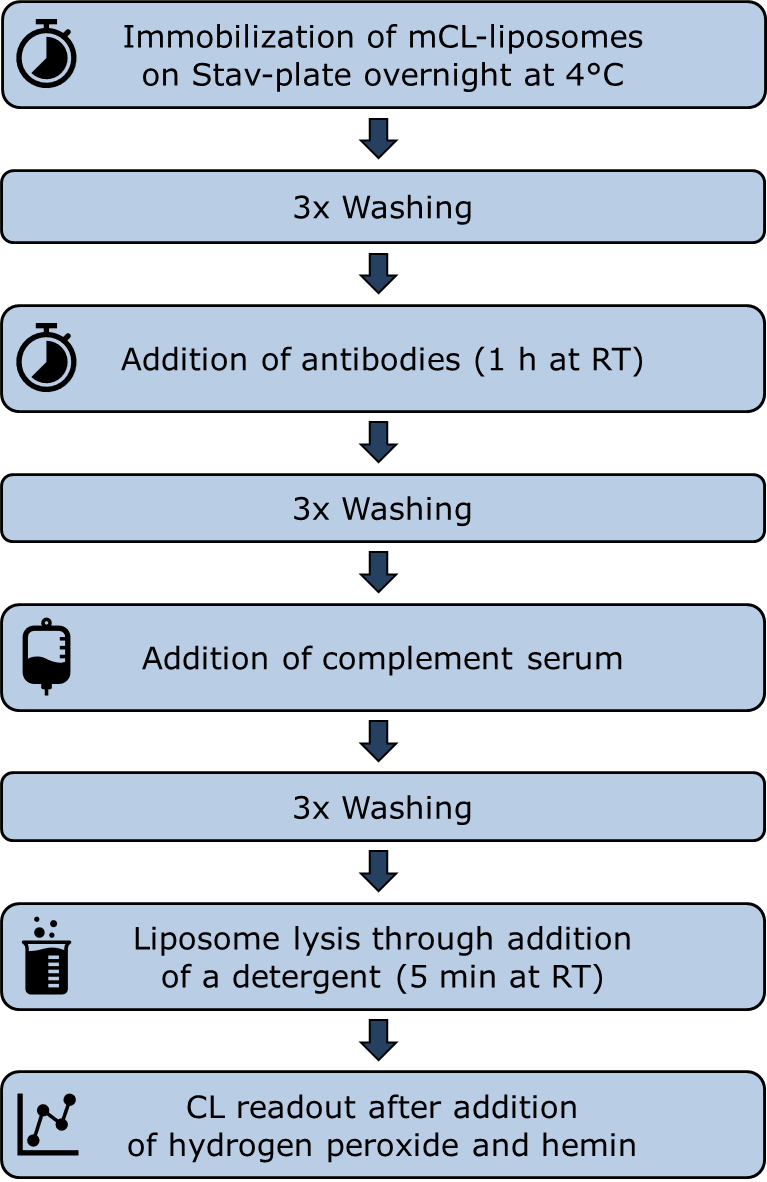


**Fig. S7** Flow chart of the heterogeneous complement assay procedure for *m*-carboxy luminol (mCL)-encapsulating liposomes.

**Fig. S8** Heterogenous complement assay of anionic, biotinylated (batch CL2) chemiluminescence liposomes (30 mM *m*‑carboxy-luminol) with 0.2 mol% anti-biotin antibody. Liposomes (50 μM total lipids) were immobilized overnight on a streptavidin-coated MTP and incubated for 1 h at 37 °C in either inactive serum or active serum (5, 10 or 25 vol% PHS as complement source). Chemiluminescence measurements were performed by adding 50 μL of 4 μM hemin and 50 μL 40 mM H_2_O_2_ in 0.01 M CBS, pH 10.5, 2 s integration time, gain 80, RH 1 mm, T = 25 °C, n = 3.


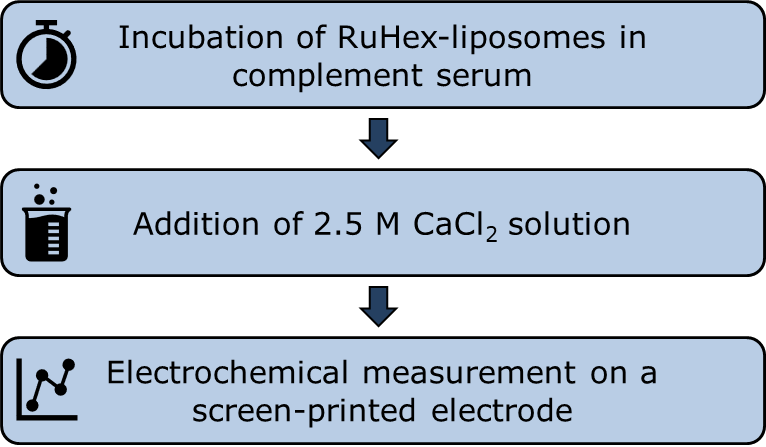


**Fig. S9** Flow chart of the assay procedure for RuHex-encapsulating liposomes.

**a**

**b**

**Fig. S10** Calibration curves of **(a)** 200 mM RuHex-loaded or **(b)** 10 mM SRB-encapsulating liposomes in 30 mM OG and 10 vol% human serum in LCB. The calibration curves were obtained by a linear fit of the data using Origin 2024. **(a)** R^2^ = 0.999; Square wave voltammograms were recorded on a DropSens screen-printed carbon electrode (DRP-110) from -0.5 V to 0.1 V. E_step_ = 4 mV, E_amp_ = 40 mV, f = 2 Hz. n = 1. **(b)** R^2^ = 0.999; λ_Ex_ = 565(8) nm and λ_Em_ =585(8) nm; gain 150, n = 3. The LOD and LOQ values were calculated by dividing the standard deviation of the y-intercept by the slope of the calibration curve and multiplying the result by 3.3 or 10, respectively.

**Fig. S11** Different combinations of anti-PEG (clone RM105 and clone 6.3) and anti‑biotin antibodies with PEG-biotin-liposomes (batch S1). A one-way ANOVA including a post-hoc Tukey test was performed for statistical analysis (p < 0.05) of the antibody combinations. Liposomes were incubated with the respective amount of antibodies (0.03 mol% clone RM105, 0.1 mol% clone 6.3, 0.3 mol% anti-biotin antibody) for 1 h at 300 rpm in 5 µL HSS. 5 vol% human serum was used as complement source (IRS45270). Fluorescence intensities were adjusted by the iaS signal and normalized to the endpoint fluorescence of the positive control. λ_Ex_ = 565(8) nm and λ_Em_ = 585(8) nm; gain 150. T = 37 °C. n = 3.

**Fig. S12** Biotin-liposomes (50 µM total lipids; batch S2) in a heterogeneous complement assay with anti-biotin and secondary anti-goat antibodies to enhance complement lysis. A one-way ANOVA including a post-hoc Tukey test was performed for statistical analysis (p < 0.05). Biotin-liposomes were immobilized for 12 h at 4 °C. The plate was washed thrice with HSS (150 µL) between each immobilization step. Antibodies (anti-biotin and anti-goat) were added separately to the plate and incubated for 1 h at 37 °C each. 10 vol% human serum was used as complement source (PHS). Fluorescence intensities were adjusted by the iaS signal and normalized to the endpoint fluorescence of the positive control. λ_Ex_ = 565(8) nm and λ_Em_ = 585(8) nm; gain 150. T = 37 °C. n = 3.

**a**

**b**

**Fig. S13** Stealthiness and triggerability of biotinylated 10 mM SRB-liposomes (10 µM total lipids; batch S12) dependent on the serum concentration. **(a)** Bare biotin-liposomes **(b)** Biotin-liposome lysis triggered by 0.5 mol% anti-biotin antibody. 1, 2, 5 or 10 vol% human serum was used as complement source (IRS45270). Fluorescence intensities were adjusted by the iaS signal and normalized to the endpoint fluorescence of the positive control. λ_Ex_ = 565(8) nm and λ_Em_ =585(8) nm; gain 150. T = 37 °C. n = 3.

**Fig. S14** Time-resolved normalized fluorescence intensities of PEGylated 10 mM SRB-liposomes (10 µM total lipids; batch S1) in a complement assay. 10 vol% human serum was used as complement source (PHS). Fluorescence measurements were carried out for 1 h at 37 °C in LCB, iaS, aS and 30 mM OG + aS. Fluorescence intensities were normalized to the endpoint fluorescence of the positive control. λ_Ex_ = 565(8) nm and λ_Em_ = 585(8) nm; gain 150. T = 37 °C. n = 3.

**a**

**b**

**c**

**d**

**Fig. S15** Stealth, non-PEGylated 10 mM SRB-liposomes (1 µM total lipids; batch S17 **(a, d)**, S10 **(b)** and S14 **(c)**) in a homogeneous complement assay. 10 vol% human serum was used as complement source (IRS45270 **(a)**, IRS41174 **(b)**, IRS35577 **(c)** and IRS31758**(d)**). Fluorescence measurements were carried out for 1 h at 37 °C in LCB, iaS, aS and 30 mM OG + aS. Fluorescence intensities were normalized to the endpoint fluorescence of the positive control. λ_Ex_ = 565(8) nm and λ_Em_ = 585(8) nm; gain 150. T = 37 °C. n = 3.


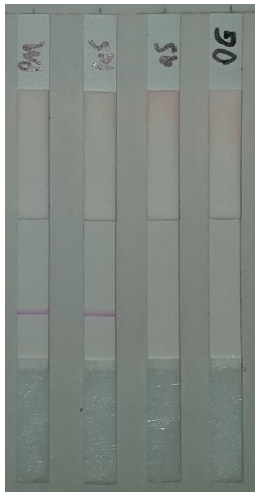


**Fig. S16** High-cholesterol 10 mM SRB-liposomes (50 μM total lipids; batch S16) in a complement assay on a lateral-flow assay after 1 h incubation at 37 °C in LCB, iaS, aS and 30 mM OG + aS. 25 vol% human serum was used as complement source (PHS). The image was taken using a Canon EOS 550D camera with a Canon EFS 18 55 mm lens from 15 cm distance (settings: ISO 100, aperture 3.5, exposure time 1/30 s, focal length 18 mm, and white balance daylight (5200 K)). The raw files were analyzed using ImageJ and the signals were background-corrected. n = 3.

**Fig. S17** Long-term storage stability study of 10 mM SRB-liposomes at 4 °C. Time-resolved fluorescence intensities of 10 mM SRB-liposomes (10 µM total lipids) with different lipid compositions (low/high-cholesterol, carboxylated, biotinylated or PEGylated; batches S1, S13, S6) in LCB, aS, iaS and 30 mM OG + aS) throughout the long-term storage stability study up to 40 months at 4 °C. 10 vol% human serum was used as complement source (PHS for 0-10 months and IRS45270 for the 40 months datapoint). λ_Ex_ = 565(5) nm and λ_Em_ = 585(5) nm; gain 150. T = 37 °C. n = 3.

**a**

**b**

**c**

**Fig. S18** Carboxylated 10 mM SRB-liposomes (1 µM total lipids; batch S17) in a homogeneous complement assay. 2 **(a)**, 5 **(b)** or 10 vol% **(c)** human serum was used as complement source (IRS45270). Fluorescence measurements were carried out for 1 h at 37 °C in LCB, iaS, iaS and 30 mM OG + aS. Fluorescence intensities were normalized to the endpoint fluorescence of the positive control. λ_Ex_ = 565(8) nm and λ_Em_ = 585(8) nm; gain 150. T = 37 °C. n = 3.

**Fig. S19** Long-term storage stability study of 10 mM SRB-liposomes at RT. Normalized fluorescence intensities of 10 mM SRB-liposomes (10 µM total lipids) with different lipid compositions **(a)** low-cholesterol, carboxylated (batch S13) **(b)** low-cholesterol, PEGylated (batch S1) and **(c)** high-cholesterol, biotinylated (batch S6) in LCB, aS or iaS throughout the long-term storage stability study up to 15 months at RT. 10 vol% human serum was used as complement source (PHS). Fluorescence intensities were normalized to the endpoint fluorescence of the positive control. λ_Ex_ = 565(5) nm and λ_Em_ = 585(5) nm; gain 150. T = 37 °C. n = 3.

**a**

**b**

**c**

**Fig. S20** Long-term storage stability study of 10 mM SRB-liposomes at RT. Time-resolved fluorescence intensities of 10 mM SRB-liposomes (10 µM total lipids) with different lipid compositions (low/high-cholesterol, carboxylated, biotinylated or PEGylated; batches S1, S13, S6) in LCB, aS, iaS and 30 mM OG + aS) throughout the long-term storage stability study up to 15 weeks at RT. 10 vol% human serum was used as complement source (PHS). Initial day 0 measurements are shown in Fig. S15. λ_Ex_ = 565(5) nm and λ_Em_ = 585(5) nm; gain 150. T = 37 °C. n = 3.

**a**

**b**

**c**

**Fig. S21** Long-term storage stability study of 10 mM SRB-liposomes at 37 °C. Normalized fluorescence intensities of 10 mM SRB-liposomes (10 µM total lipids) with different lipid compositions **(a)** low-cholesterol, carboxylated (batch S13) **(b)** low-cholesterol, PEGylated (batch S1) and **(c)** high-cholesterol, biotinylated (batch S6) in LCB, aS or iaS throughout the long-term storage stability study up to 12 weeks at 37 °C. 10 vol% human serum was used as complement source (PHS). Fluorescence intensities were normalized to the endpoint fluorescence of the positive control. λ_Ex_ = 565(5) nm and λ_Em_ = 585(5) nm; gain 150. T = 37 °C. n = 3.

**Fig. S22** Long-term storage stability study of 10 mM SRB-liposomes at 37 °C. Time-resolved fluorescence intensities of 10 mM SRB-liposomes (10 µM total lipids) with different lipid compositions (low/high-cholesterol, carboxylated, biotinylated or PEGylated; batches S1, S13, S6) in LCB, aS, iaS and 30 mM OG + aS) throughout the long-term storage stability study up to 12 weeks at 37 °C. 10 vol% human serum was used as complement source (PHS). Initial day 0 measurements are shown in Fig. S15. λ_Ex_ = 565(5) nm and λ_Em_ = 585(5) nm; gain 150. T = 37 °C. n = 3.
